# Supplementary material for: Quantifying the factors limiting rate performance in battery electrodes
Source: Nat Commun. 2019 Apr 29;10:1933. doi: 10.1038/s41467-019-09792-9 (PMC6488605; doi:10.1038/s41467-019-09792-9)
Supplement: Supplementary file 1 — Supplementary Information [file 41467_2019_9792_MOESM1_ESM.pdf]

Supplementary Information for

Quantifying the factors limiting rate-performance in battery electrodes

Ruiyuan Tian *et al.*

Supplementary Figures

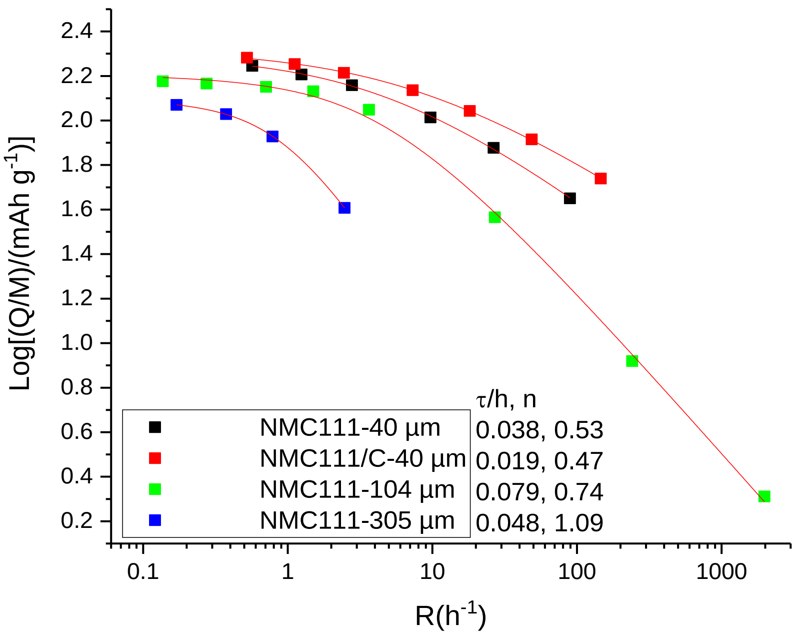

Supplementary Figure 1. NMC111 fits. (Supplementary Table 2, row 1-3)

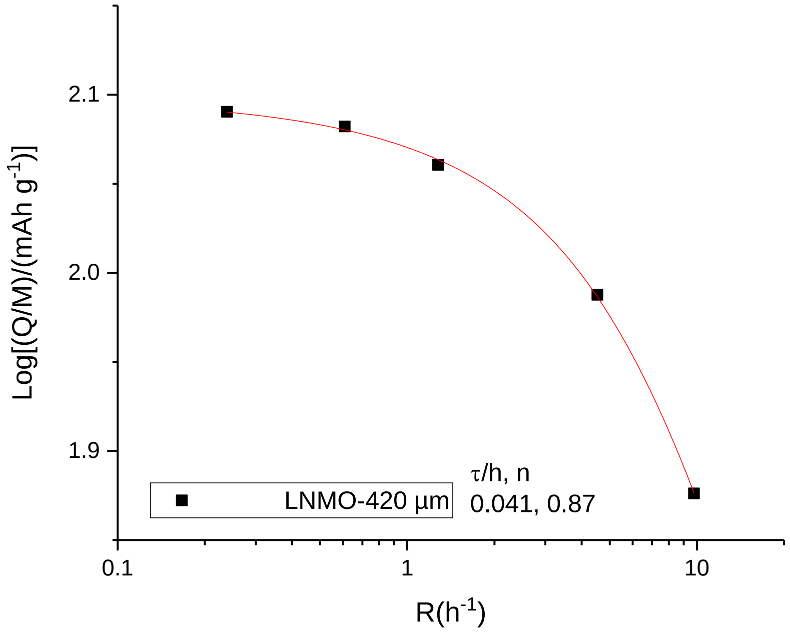

Supplementary Figure 2. $LiNi_{0.5}Mn_{1.5}O_4$-fits. (Supplementary Table 2, row 4)

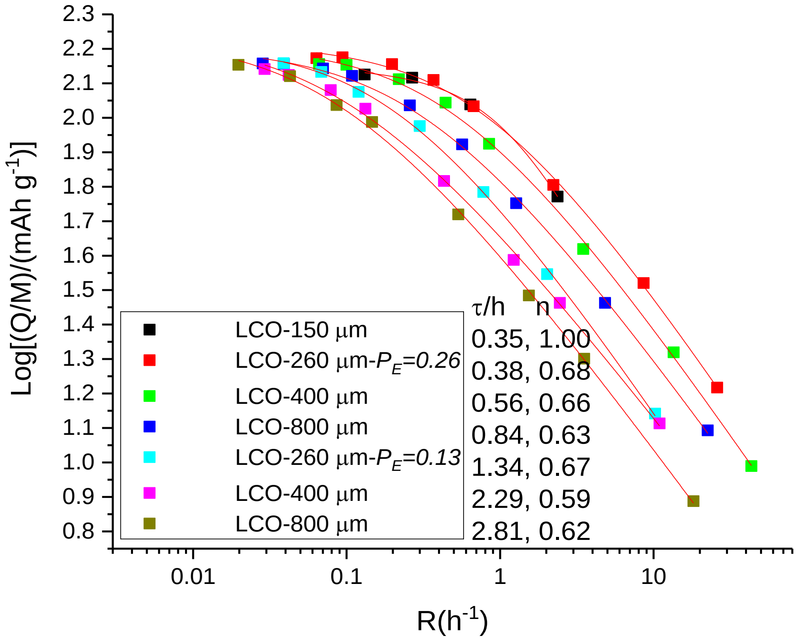

Supplementary Figure 3. $LiCoO_2$ fits. (Supplementary Table 2, row 5−6)

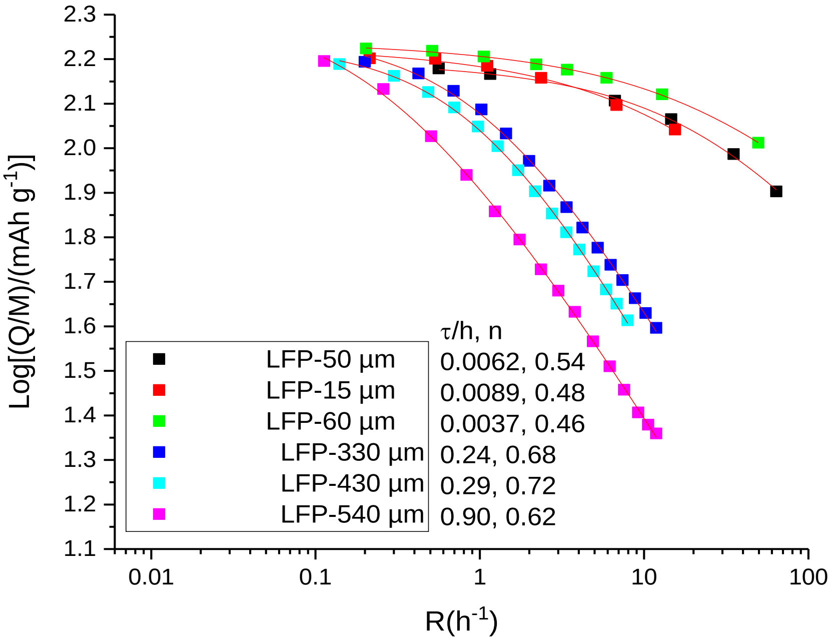

Supplementary Figure 4. $LiFePO_4$ fits. (Supplementary Table 2, row 7−10)

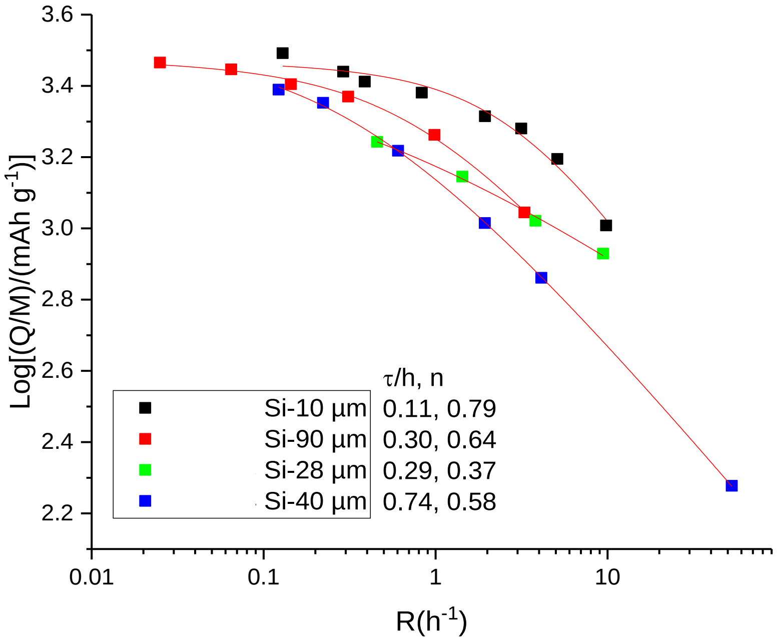

Supplementary Figure 5. Si fits. (Supplementary Table 2, row 11−14)

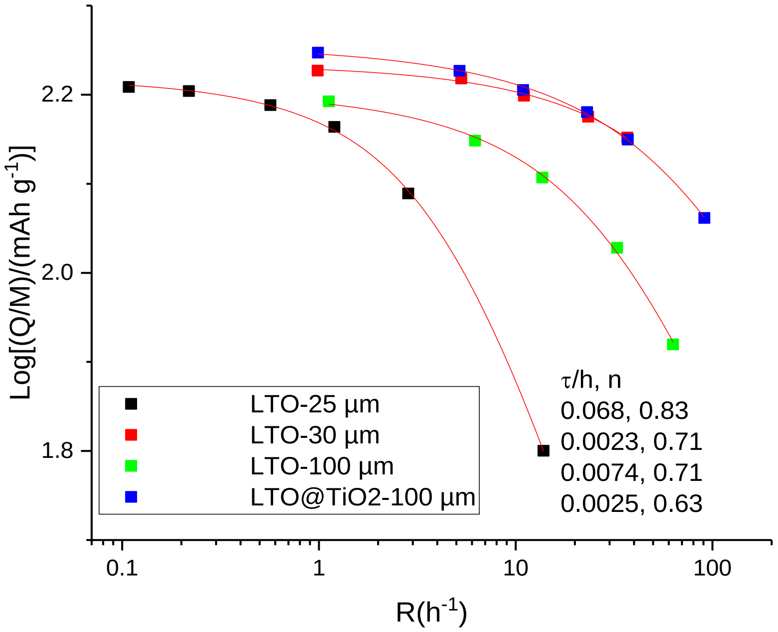

Supplementary Figure 6. $Li_4Ti_5O_{12}$ fits. (Supplementary Table 2, row 15−17)

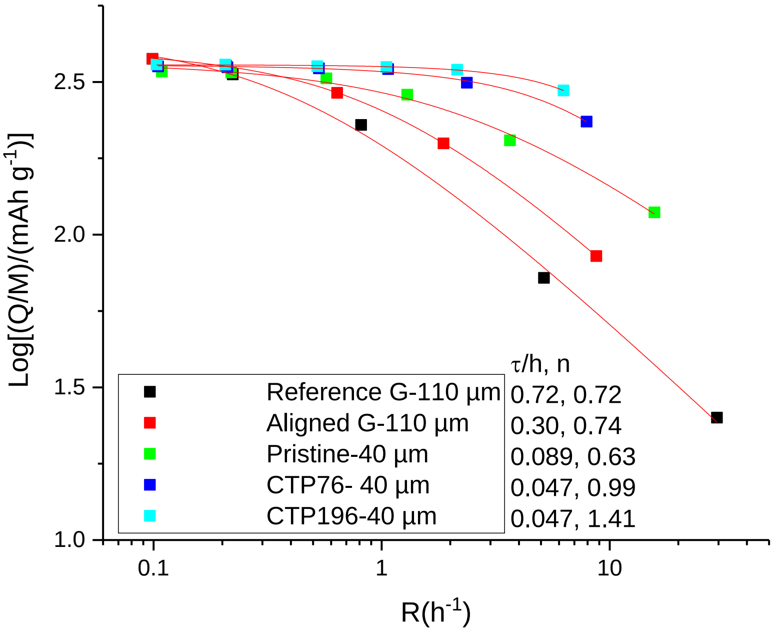

Supplementary Figure 7. Graphite fits. (Supplementary Table 2, row 18−19)

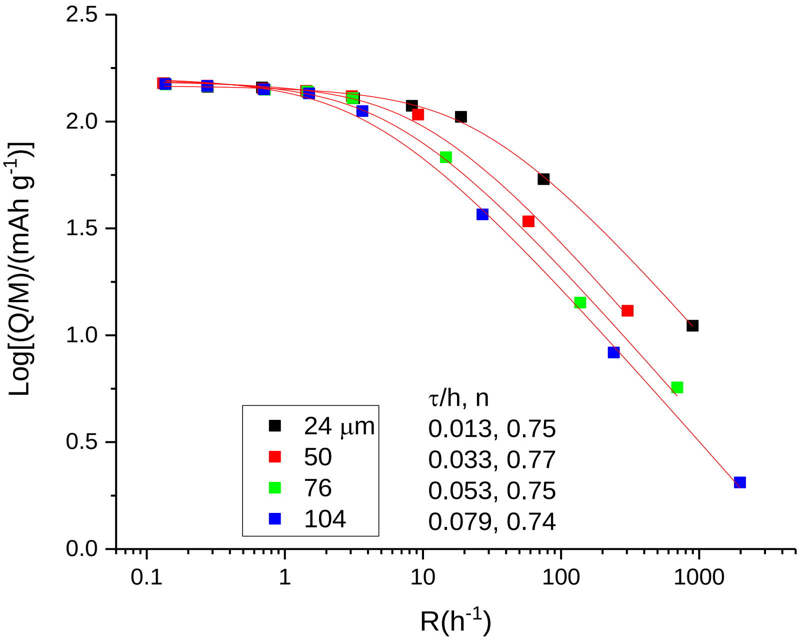

Supplementary Figure 8. NMC fits. (Supplementary Table 3, row 1)

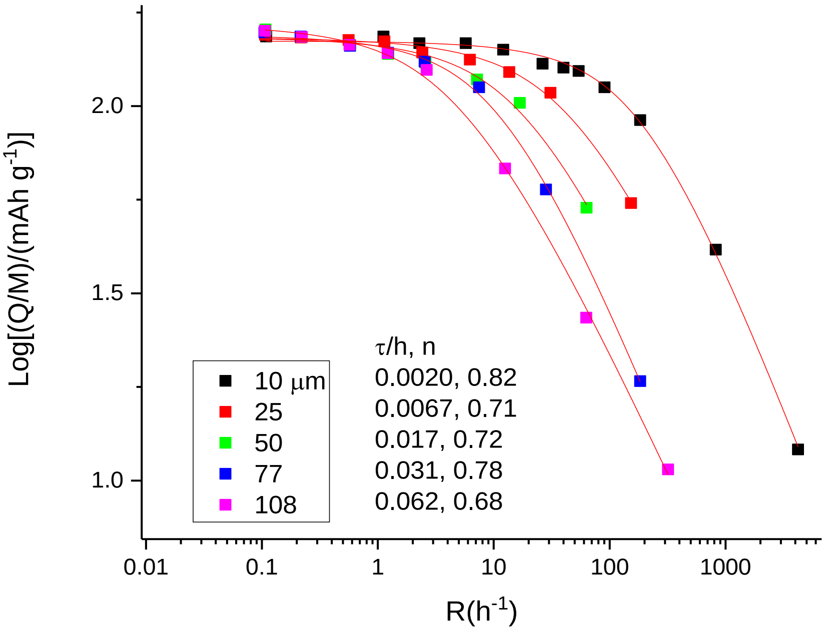

Supplementary Figure 9. LFP fits. (Supplementary Table 3, row 1)

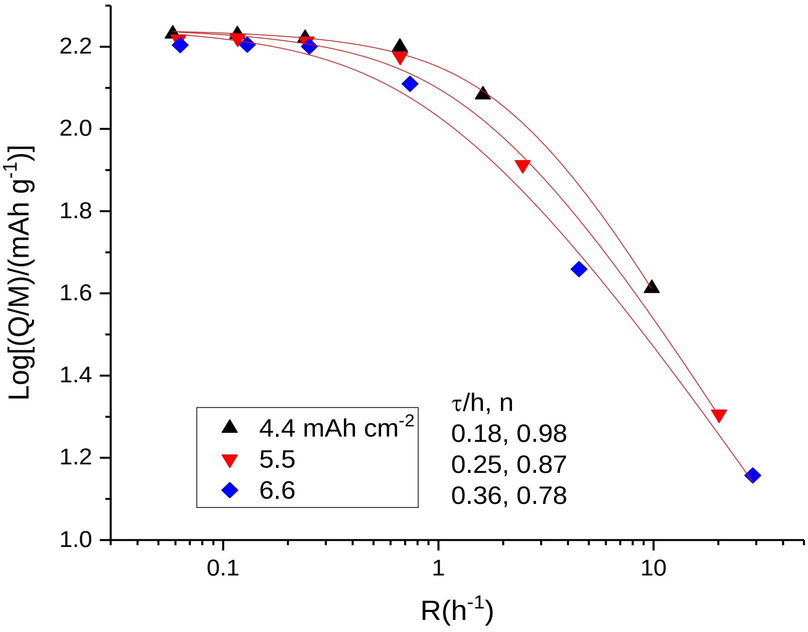

Supplementary Figure 10. Graphite/NMC full cell. N.B. The data sets associated with the two thinnest electrodes displayed very little change in Q/M with R leading to unreliable fits. (Supplementary Table 3, row 2)

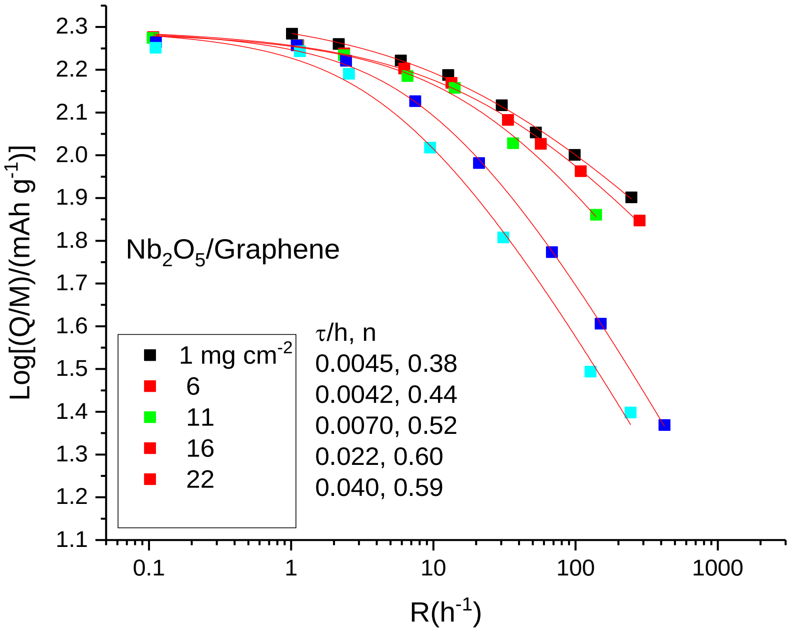

Supplementary Figure 11. Niobia/graphene fits. (Supplementary Table 3, row 3)

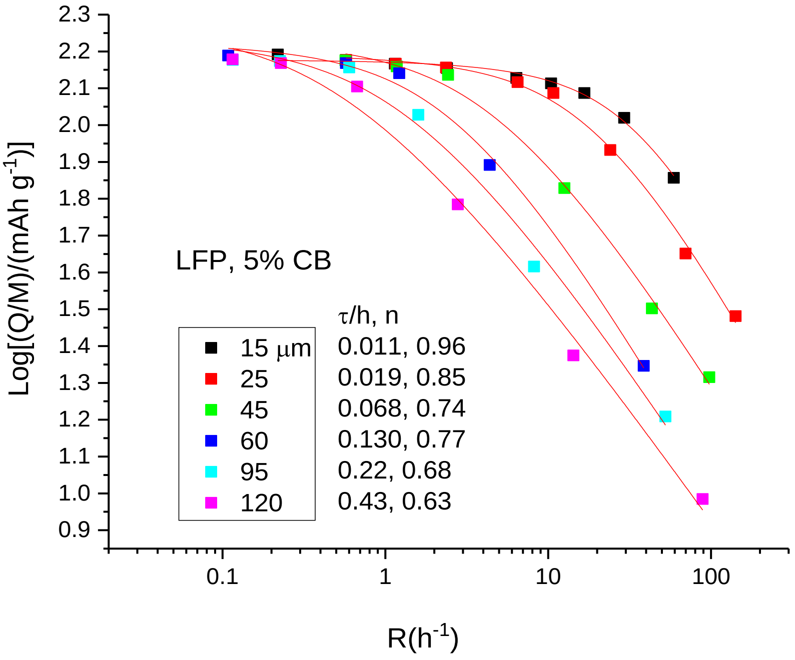

Supplementary Figure 12. LFP, 5% AB (acetylene black) fits. (Supplementary Table 3, row 4)

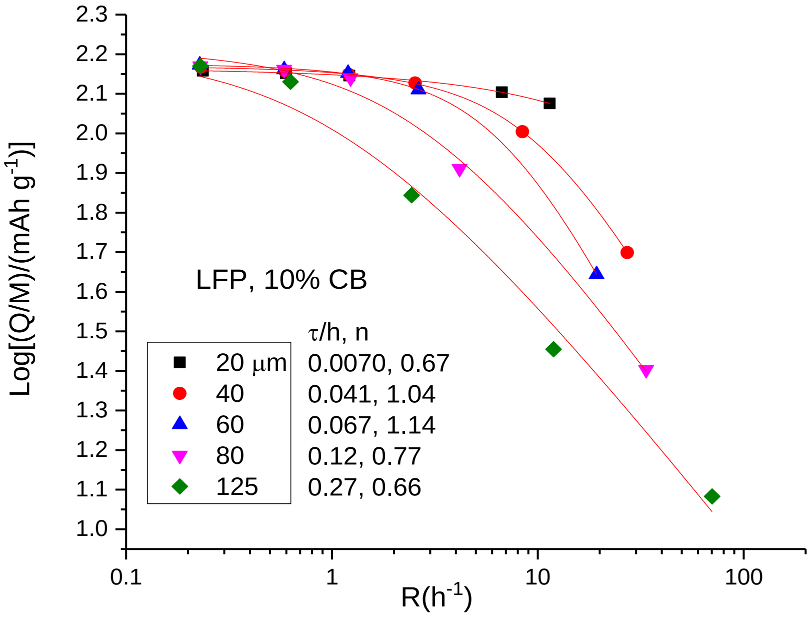

Supplementary Figure 13. LFP, 10% CB (acetylene black) fits. (Supplementary Table 3, row 4)

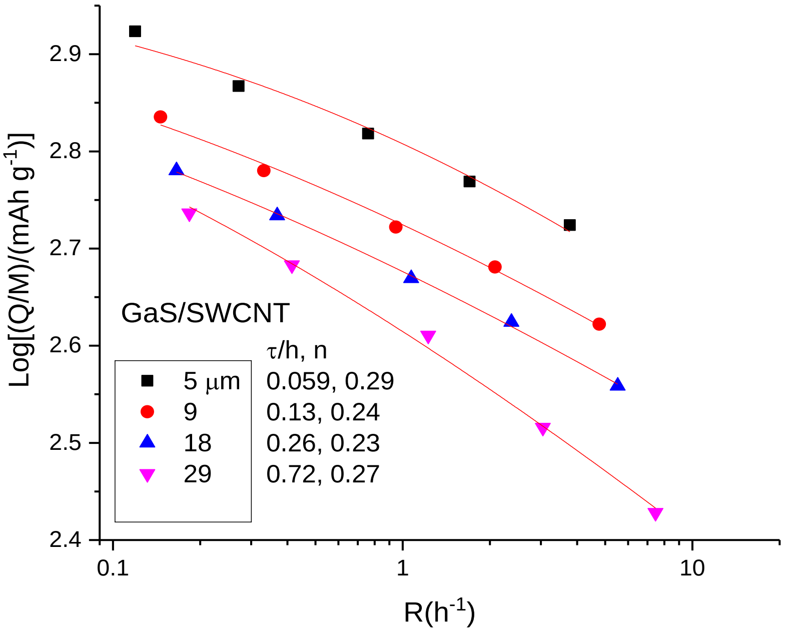

Supplementary Figure 14. GaS/SWCNT fits. (Supplementary Table 3, row 5)

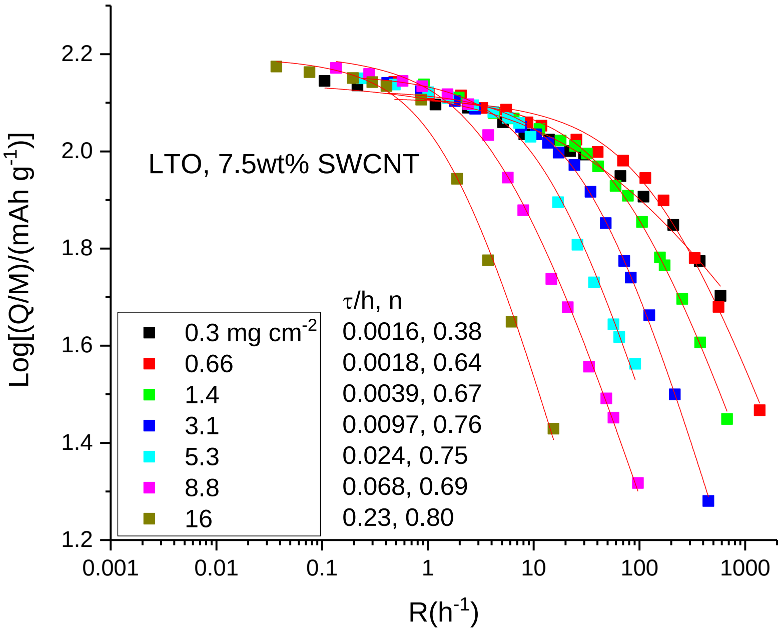

Supplementary Figure 15. This work, LTO/SWCNT (7.5%), fits. (Supplementary Table 3, row 6)

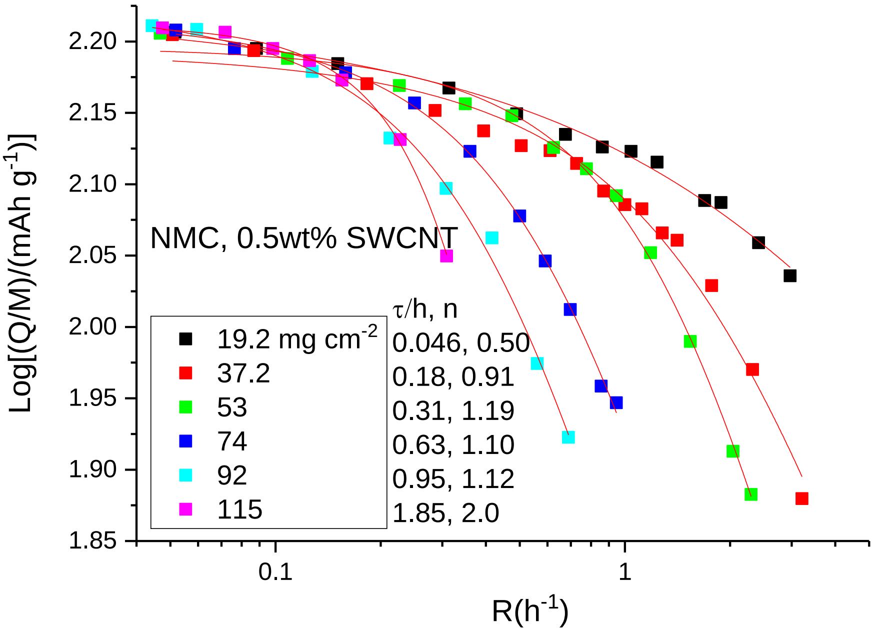

Supplementary Figure 16. This work, NMC/SWCNT (0.5%). (Supplementary Table 3, row 7)

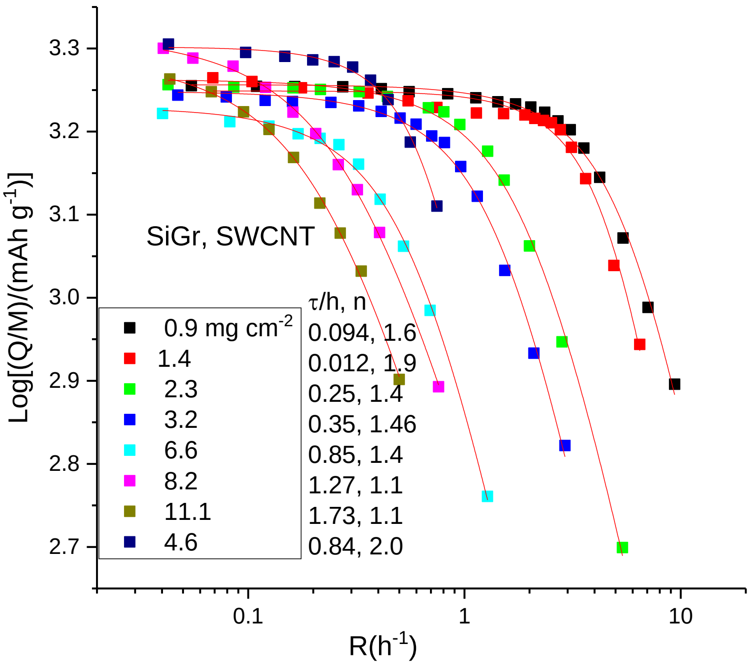

Supplementary Figure 17. This work, SiGr/SWCNT. (Supplementary Table 3, row 8)

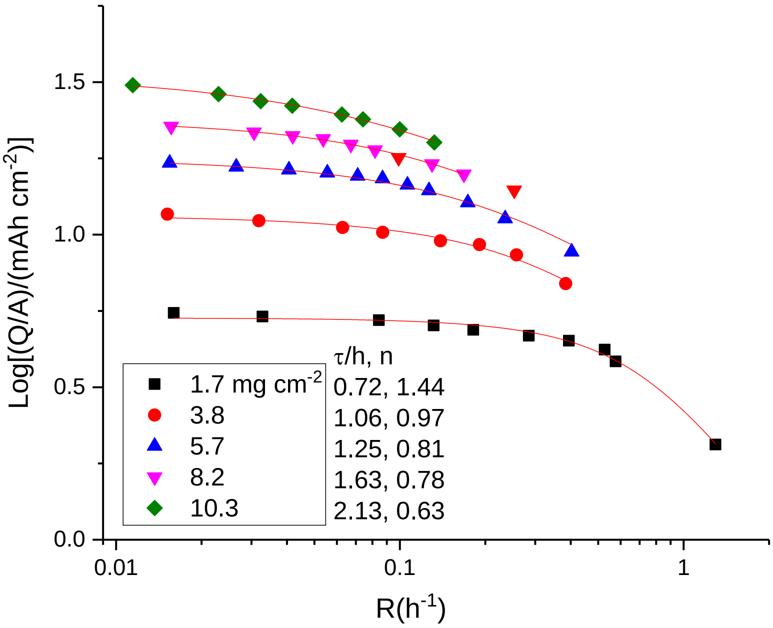

Supplementary Figure 18. This work, micron-Si/SWCNT. (Supplementary Table 3, row 9)

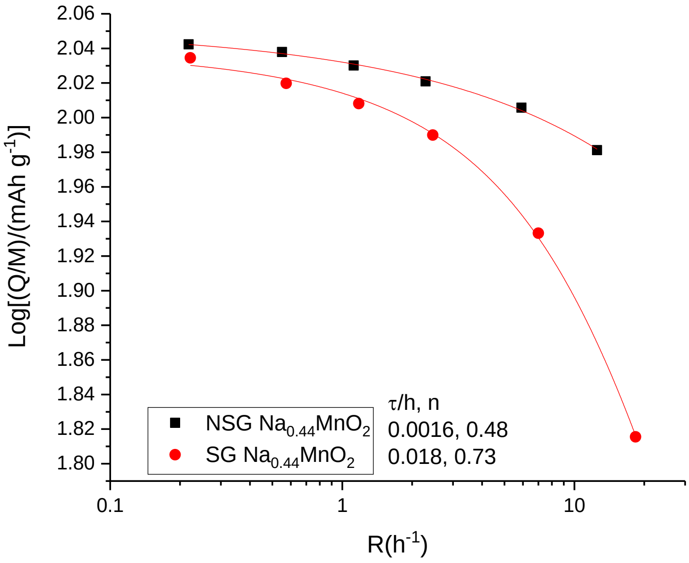

Supplementary Figure 19. $Na_{0.44}MnO_2$ fits. (Supplementary Table 4, row 1)

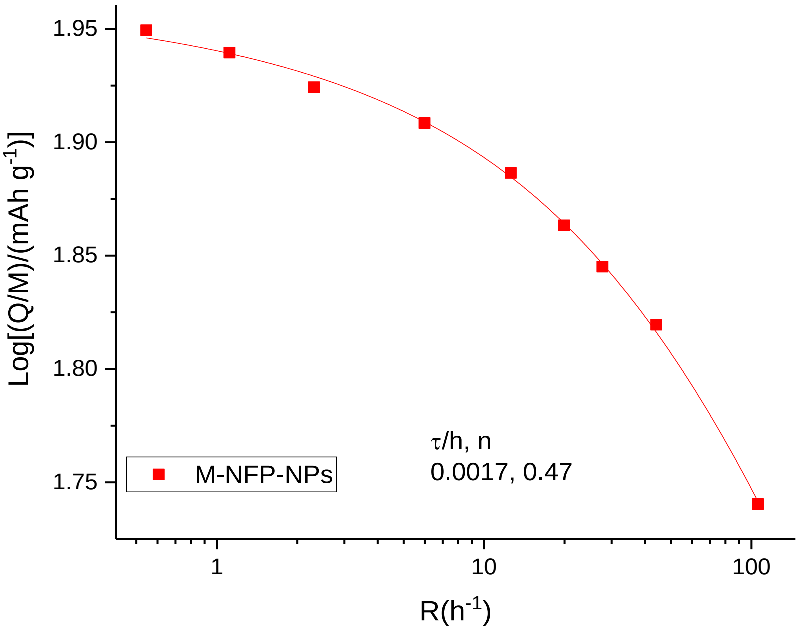

Supplementary Figure 20. $Na_2FeP_2O_7$ fits. (Supplementary Table 4, row 2)

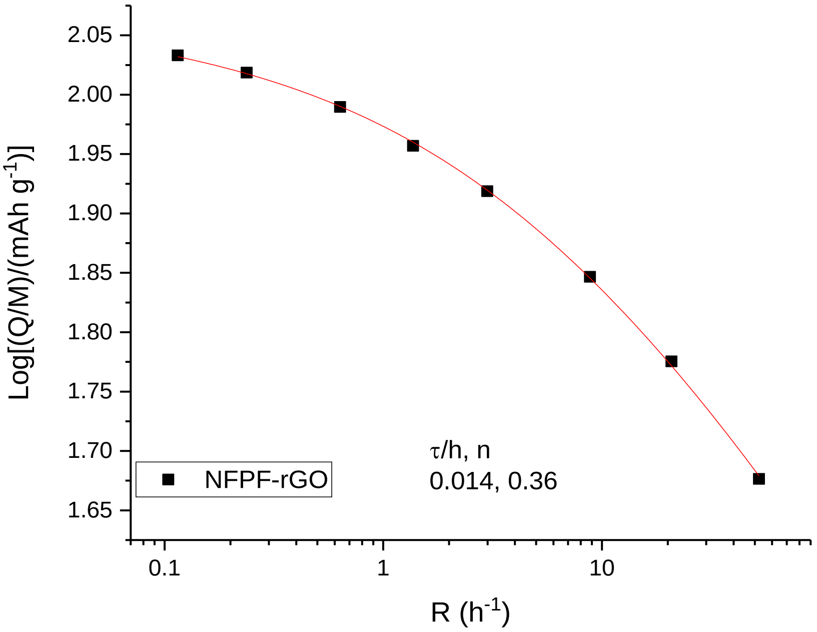

Supplementary Figure 21. Na_2FePO_4F fits. (Supplementary Table 4, row 3)

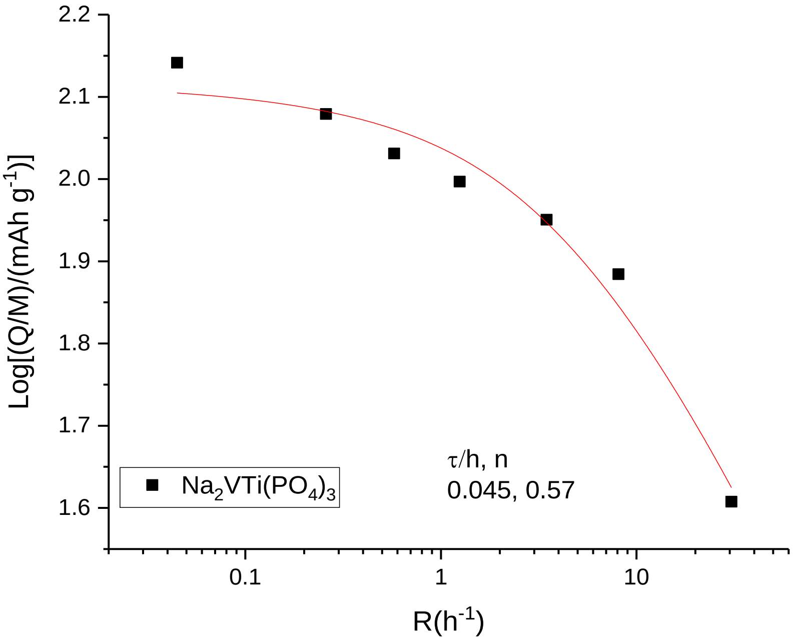

Supplementary Figure 22. $Na_2VTi(PO_4)_3$ fits. (Supplementary Table 4, row 4)

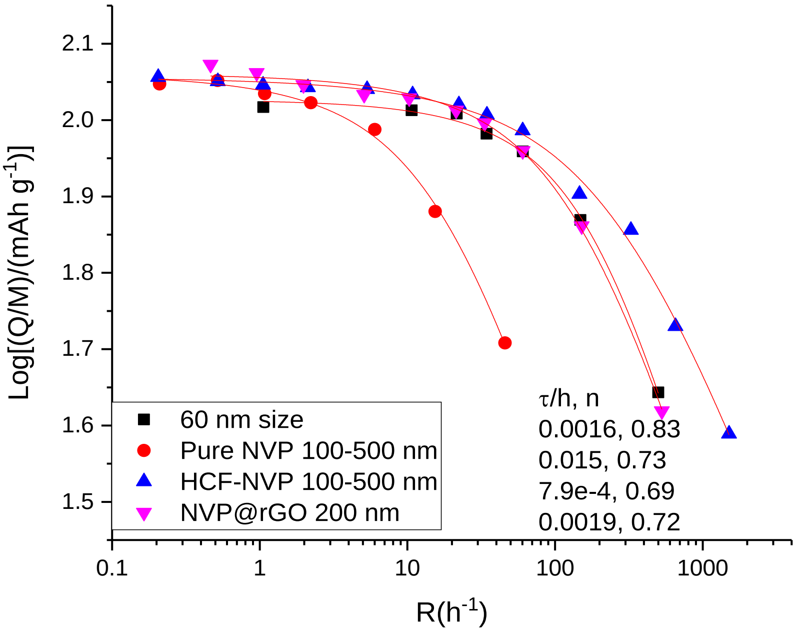

Supplementary Figure 23. $Na_3V_2(PO_4)_3$ fits. (Supplementary Table 4, row 5−7)

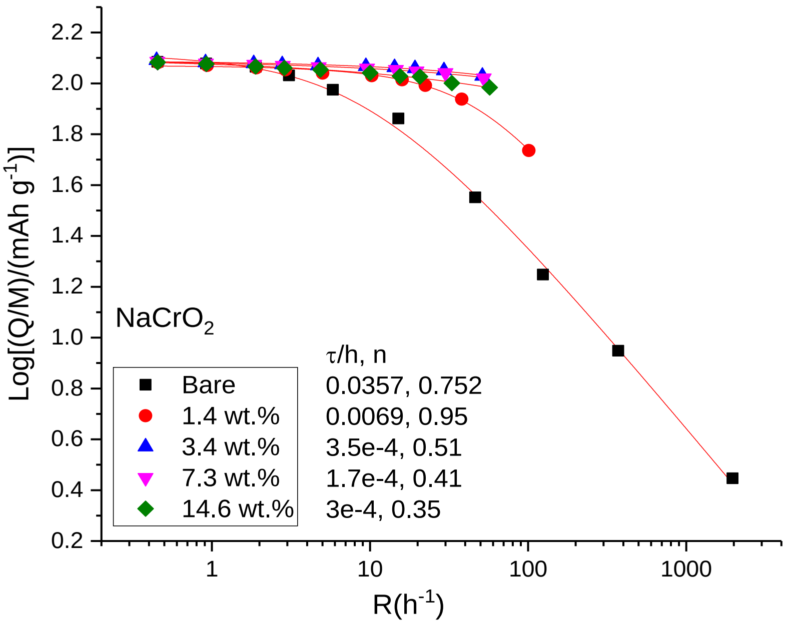

Supplementary Figure 24. $NaCrO_2$/C fits. (Supplementary Table 4, row 8)

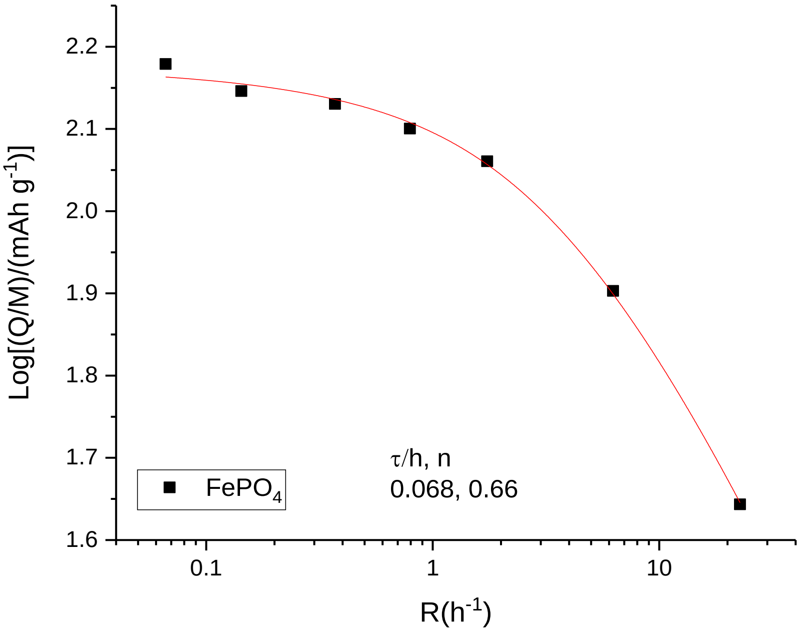

Supplementary Figure 25. $FePO_4$ fits. (Supplementary Table 4, row 9)

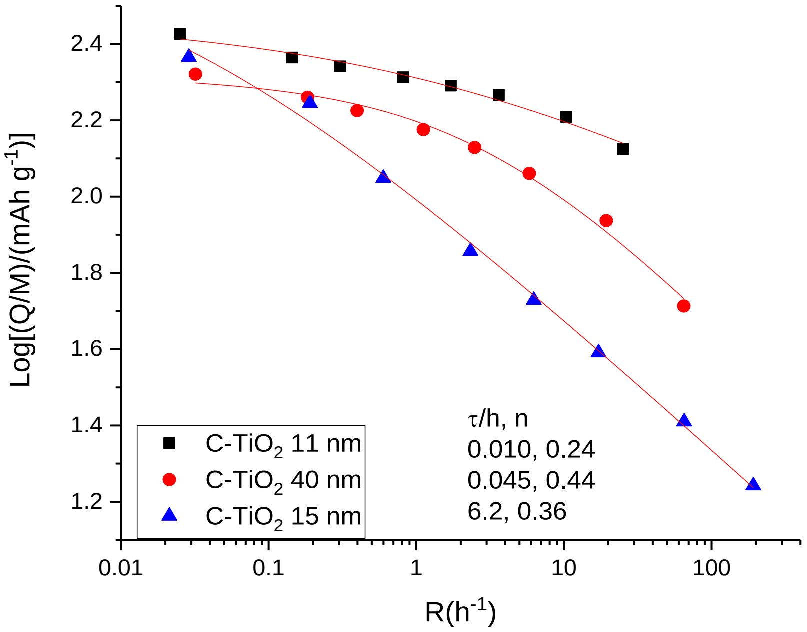

Supplementary Figure 26. TiO_2 fits. (Supplementary Table 4, row 10)

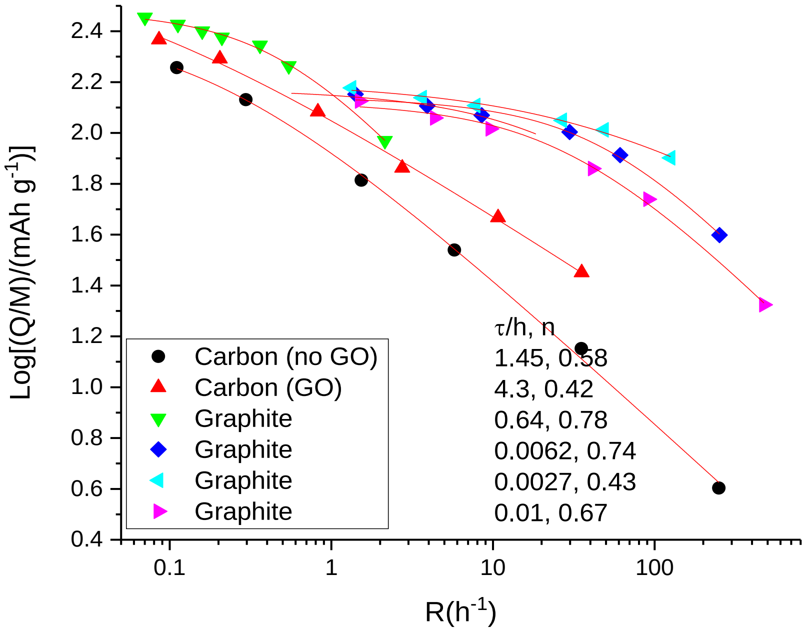

Supplementary Figure 27. Carbon/Graphite fits. (Supplementary Table 4, row 11−13)

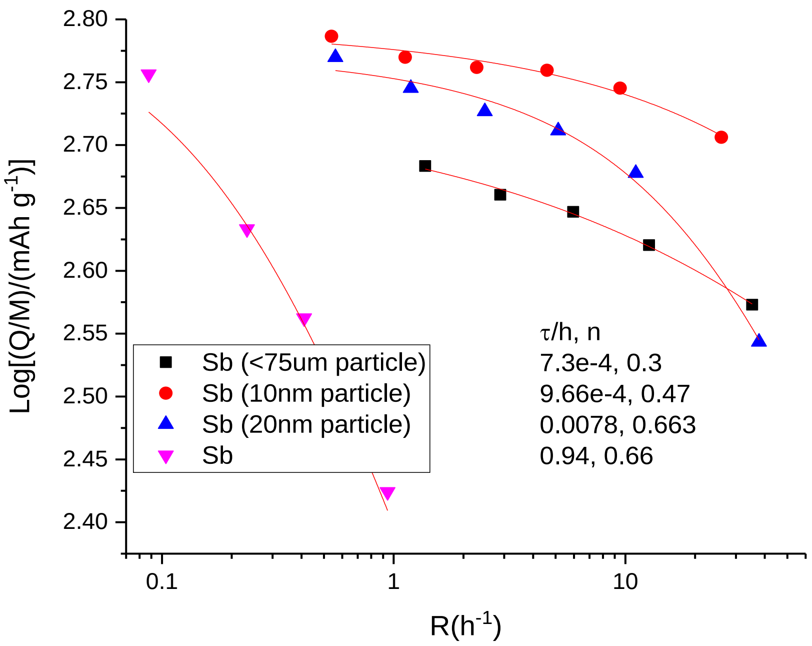

Supplementary Figure 28. Sb fits. (Supplementary Table 4, row 14–15)

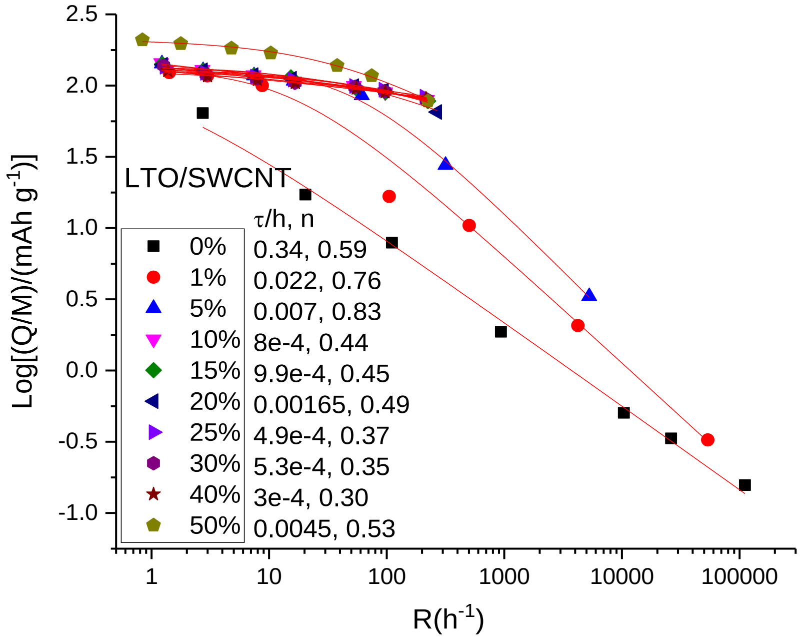

Supplementary Figure 29. LTO/SWCNT fits. (Supplementary Table 5, row 1)

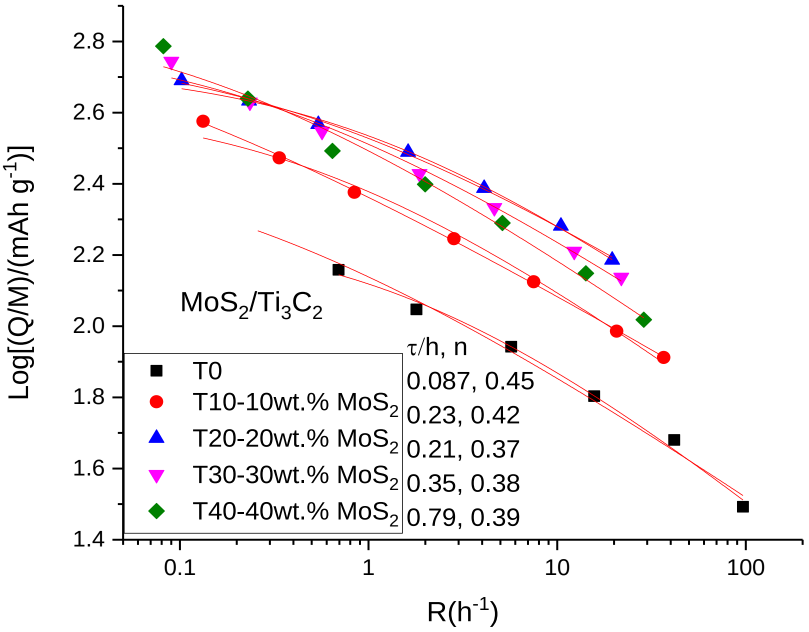

Supplementary Figure 30. LTO/SWCNT fits. (Supplementary Table 5, row 2)

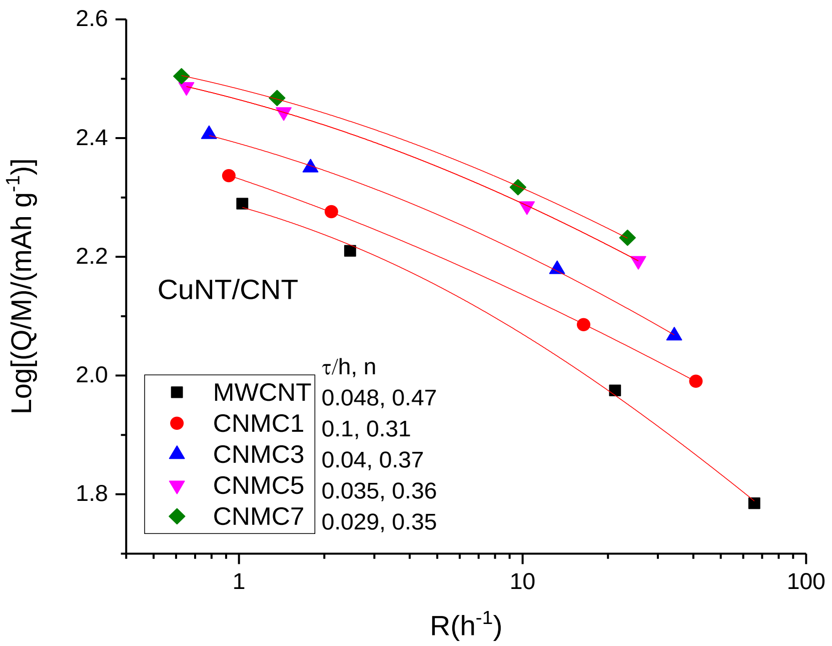

Supplementary Figure 31. CuNT/CNT fits. (Supplementary Table 5, row 3)

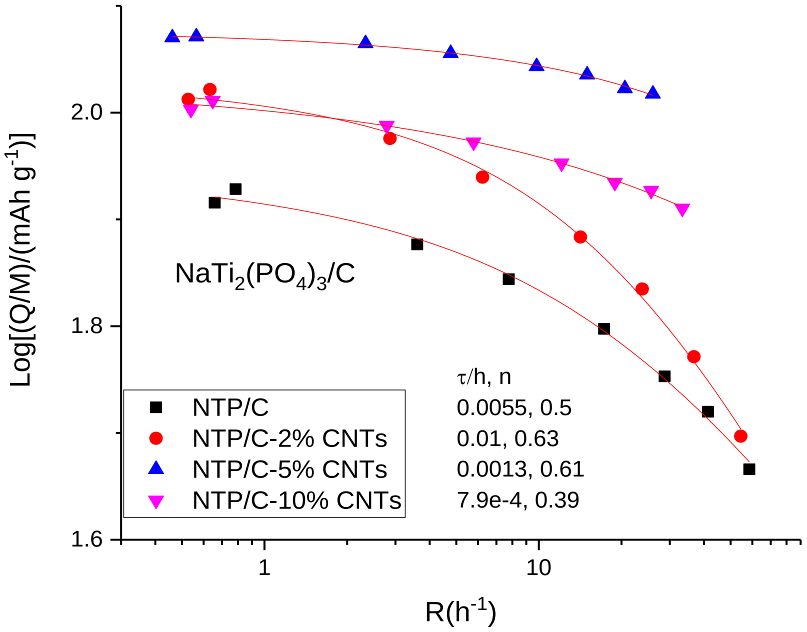

Supplementary Figure 32. $NaTi_2(PO_4)_3/C$ fits. (Supplementary Table 5, row 4)

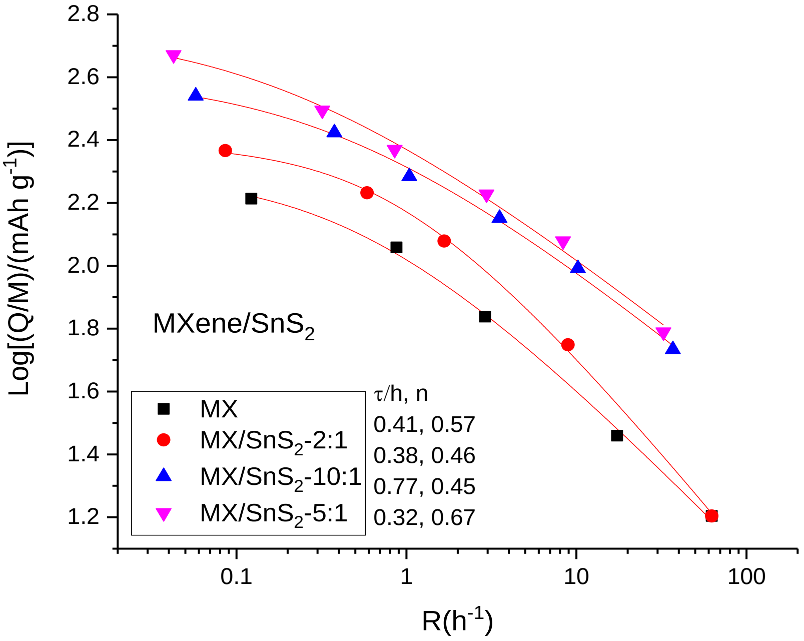

Supplementary Figure 33. MXene/SnS_2 fits. (Supplementary Table 5, row 5)

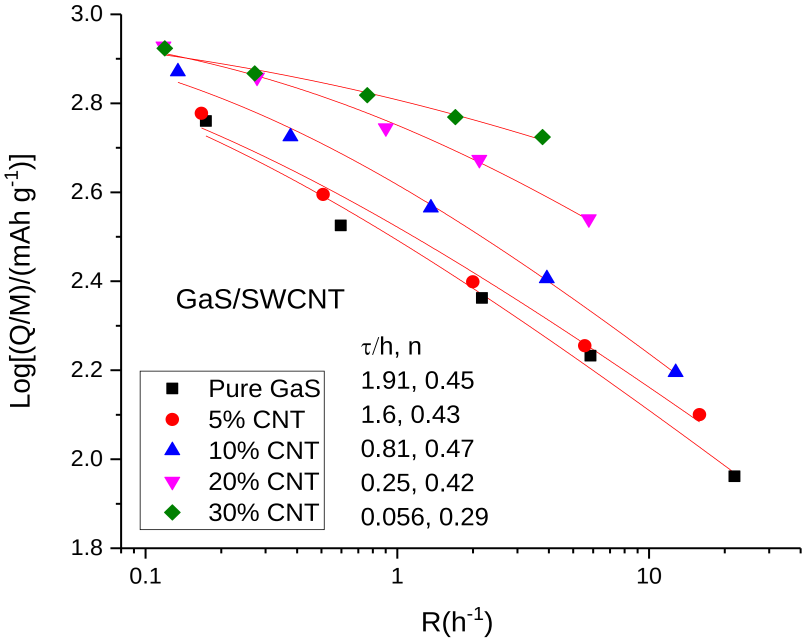

Supplementary Figure 34. GaS/SWCNT fits. (Supplementary Table 5, row 6)

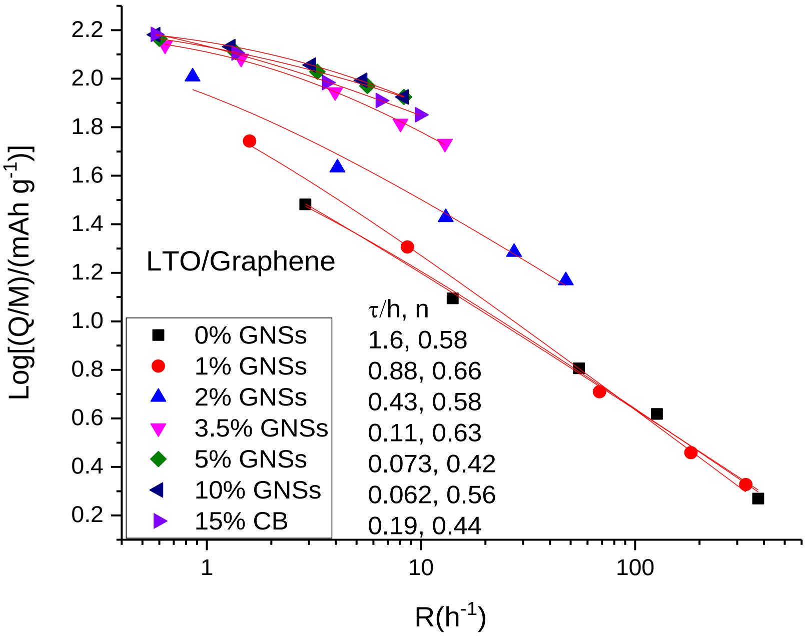

Supplementary Figure 35. LTO/Graphene fits. (Supplementary Table 5, row 8)

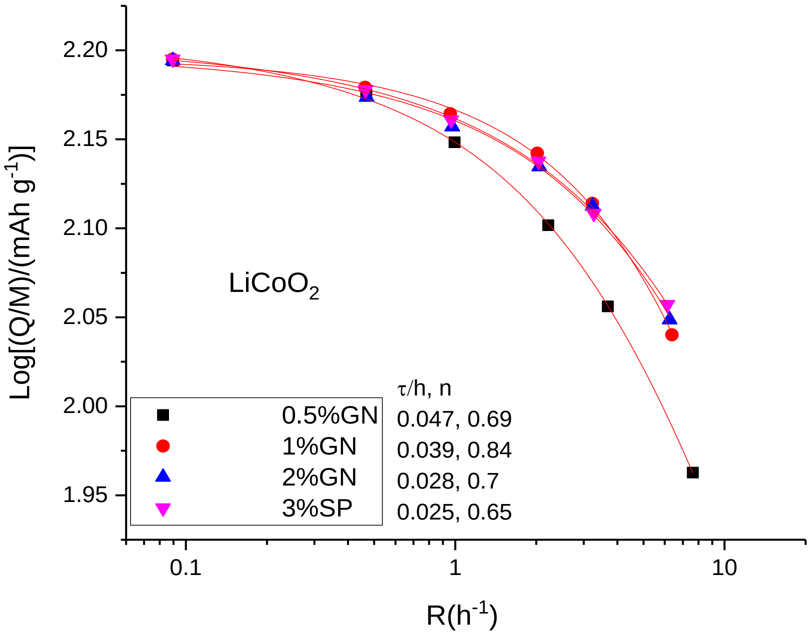

Supplementary Figure 36. LCO/Graphene fits. (Supplementary Table 5, row 9)

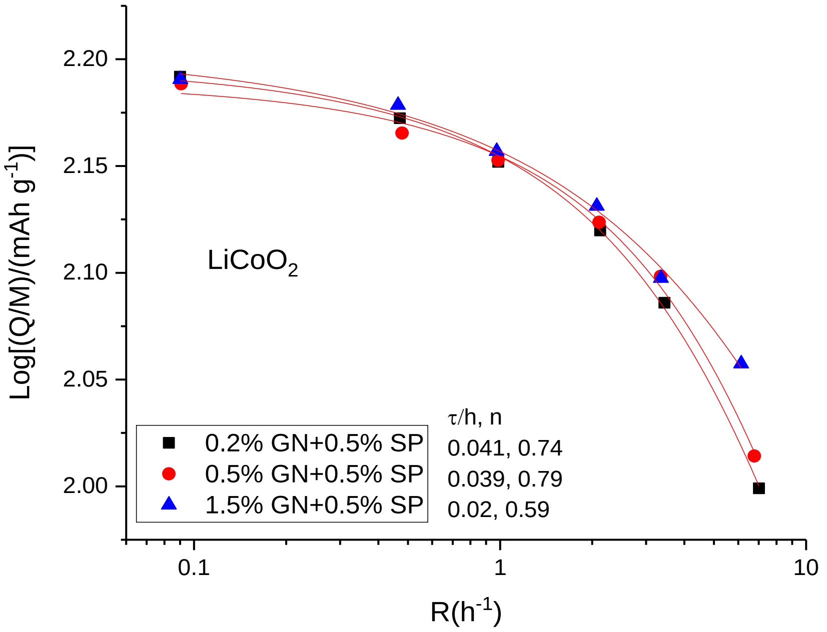

Supplementary Figure 37. LCO/Graphene/0.5wt.% Super P fits. (Supplementary Table 5, row 9)

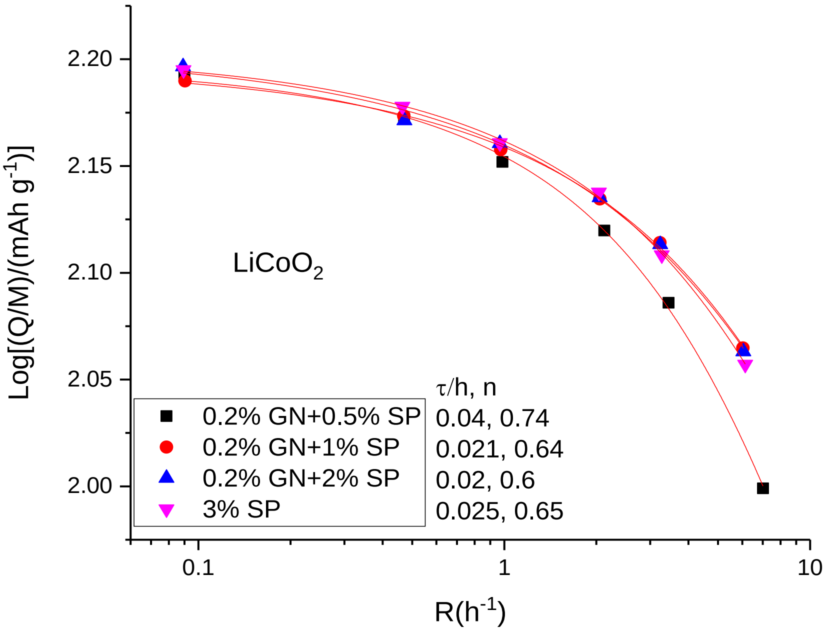

Supplementary Figure 38. LCO/0.2wt.%Graphene/Super P fits. (Supplementary Table 5, row 9)

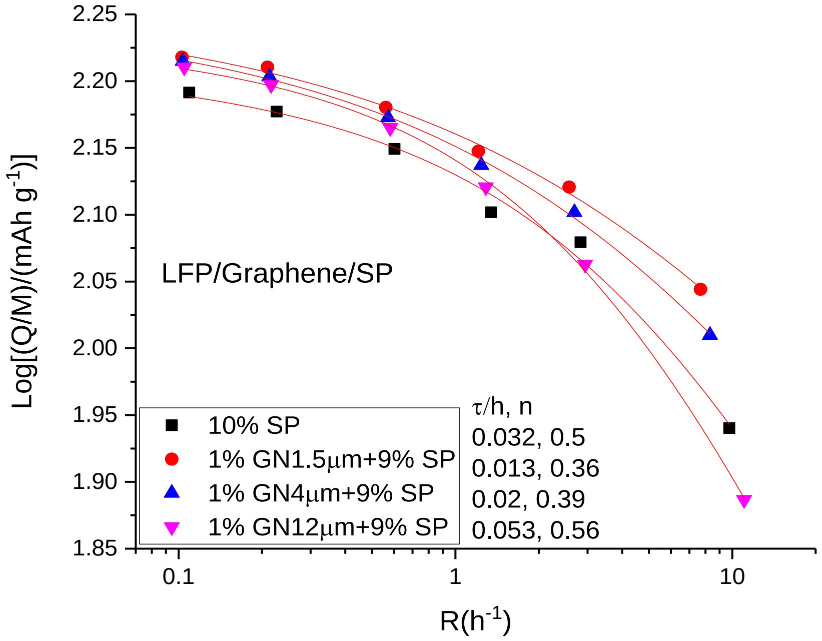

Supplementary Figure 39. LFP/Graphene/Super P fits. (Supplementary Table 5, row 10)

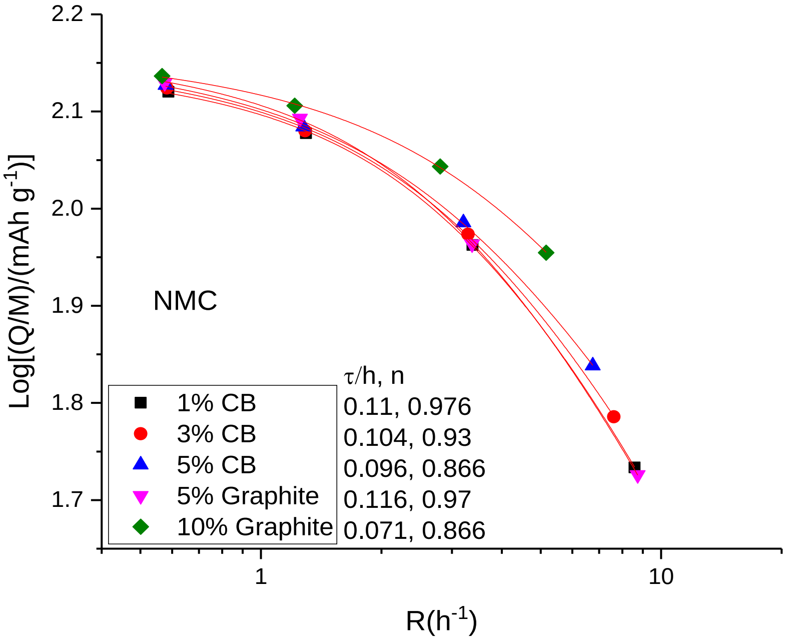

Supplementary Figure 40. NMC/Graphite/CB fits. (Supplementary Table 5, row 11)

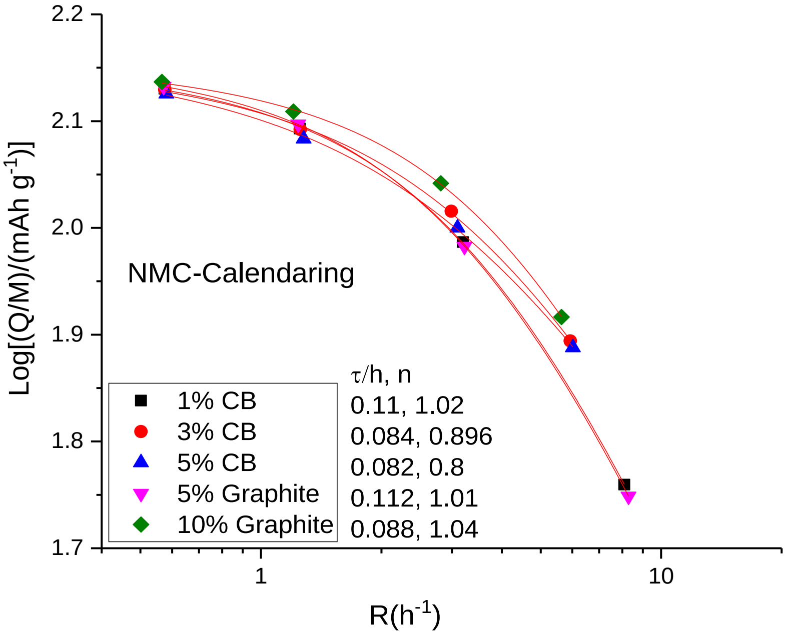

Supplementary Figure 41. NMC/Graphite/CB with Calendaring fits. (Supplementary Table 5, row 11)

Supplementary Tables

Supplementary Table 1. Physical percolation properties from references (figure 3 main text).

	$(C_{eff}/V)/\sigma_M$	σ_0/σ_M	s	β_1	Q_V	$C_{V,eff}$	σ_M	σ_0
units	s m^{-2}	-	-	s m^{-2}	mAh m^{-3}	F m^{-3}	S m^{-1}	S m^{-1}
J Zhang	2.7×10^{14}	1060	3	2×10^{9}	2.0×10^{9}	5.6×10^{10}	1.0×10^{-4}	0.11
J Coelho	10^{15}	4000	1.4	2×10^{9}	2.8×10^{8}	7.8×10^{9}	3.9×10^{-6}	1.6×10^{-2}
B Zhang	5.5×10^{13}	4×10^{6}	3.5	2.2×10^{12}	5.3×10^{8}	1.5×10^{10}	1.3×10^{-4}	5.4×10^{2}
Yu	3.3×10^{11}	1.2×10^{6}	2.7	2.3×10^{9}	4.8×10^{8}	1.3×10^{10}	2.0×10^{-2}	2.5×10^{4}

Cohort I: Lithium ion batteries

Supplementary Table 2. Literature details for Cohort IA

Row No.	Electrode	Electrode composition	Mass loading (M_{Total}/A and M_{AM}/A)	Particle size, morphology or density	Electrode thickness	Ref.
**NMC111 (1C = 200 mA g^{-1})**						
1	Carbon coated NMC111	AM: PTFE: C = 80: 5: 15	6.3 mg cm^{-2} (AM: 5 mg cm^{-2})	Particle size: 50−200 nm	≈ 40 µm	1
				Particle size: 150 nm with 10 nm carbon coating		
2	Slurry-casted NMC111 electrode	NMC111: CB: PVDF = 85: 7: 8	28 mg cm^{-2} (NMC: 24 mg cm^{-2})	Particle size: 6−8 µm	104 µm	2
3	Slurry-casted NMC111 electrode	NMC111: CB: graphite: PVDF =90: 3: 4: 3 Full Pouch bag cells	82 mg cm^{-2} (NMC: 73.8 mg cm^{-2})	Commercial NMC (BASF)	305 µm	3
**$LiNi_{0.5}Mn_{1.5}O_4$ (1C = 147 mA g^{-1})**						
4	Electrospray/ spinning of LMNO	LMNO: MWCNT: PAN = 72: 3.6: 24.4	80 mg cm^{-2} (LMNO: 57 mg cm^{-2})	Particle size: 100−500 nm	420 µm	4
**$LiCoO_2$ (1C = 140 mA g^{-1})**						
5	$LiCoO_2$/CNT free-standing film by vacuum−filtration (CNT as substrate)	LCO: CB: PVDF = 77.5: 12.5: 10	16.1 mg cm^{-2} (LCO: 12.5 mg cm^{-2})	Particle size: 5−10 µm	150 µm	5
6	Mechanically pressed $LiCoO_2$ pellet electrode	Free-standing pellet	97.16 mg cm^{-2}	74% of the theoretical crystal density (5.05 g cm^{-3})	260 µm	6
			149.48 mg cm^{-2}		400 µm	
			298.96 mg cm^{-2}		800 µm	
			114.23 mg cm^{-2}	87% of the theoretical crystal density (5.05 g cm^{-3})	260 µm	
			175.74 mg cm^{-2}		400 µm	
			351.48 mg cm^{-2}		800 µm	
**$LiFePO_4$ (1C = 170 mA g^{-1})**						
7	High tap density nanoplate packed $LiFePO_4$/C microspheres	AM: AB: PTFE = 75: 20: 5 (Symmetric charge/discharge rate)	≈ 6.67 mg cm^{-2} (AM: 5 mg cm^{-2})	Secondary particle size: 0.5−2 µm Tap density for AM: 1.4 g cm^{-3}	≈50 µm	7

8	Soft-contact conductive carbon (SCC) to $LiFePO_4$	LFP: SCC: PTFE = 70: 20: 10	3.64 mg cm^{-2} (AM: 2.55 mg cm^{-2})	Particle size: 50 nm	15 µm	8
9	Vacuum-filtrated CNF−CNT−$LiFePO_4$ film	LFP: CNT: CNF = 80: 15: 5	5.5 mg cm^{-2} (LFP: 4.4 mg cm^{-2})	Particle size: 200−700 nm	≈ 60 µm	9
10	Slurry-coating $LiFePO_4$ onto porous metal foam substrate	LFP/C: CB: PVDF = 75: 15: 10	43.3 mg cm^{-2} (AM: 32.5 mg cm^{-2})	Particle size: Unknown<br><br>Electrode density: 1.35 g cm^{-3}	330 µm	10
			62 mg cm^{-2} (AM: 46.5 mg cm^{-2})		430 µm	
			75.1 mg cm^{-2} (AM: 56.3 mg cm^{-2})		540 µm	
**Si (1C = 3500 mA g^{-1})**						
11	PEDOT: PSS + CMC binder	Si: AB: (PEDOT: PSS): CMC =70:10:10:10	1.5 mg cm^{-2} (Si: 1.05 mg cm^{-2})	Particle size: 50–100 nm	10 µm	11
12	CVD growth of Si nanowires	Si film *via* CVD	Si: 2.47 mg cm^{-2}	Nanowires with D ≈ 300nm	90 µm	12
13	Si-graphene composite by vacuum filtration	Si: graphene = 62: 38	3.7 mg cm^{-2} (Si: 2.3 mg cm^{-2})	Particle size: ≈100 nm	28 µm	13
14	Si nanowire-graphene composites	Si: 70−80%	≈ 8 mg cm^{-2} (Si: 6 mg cm^{-2})	Nanowires with D= 10–100 nm	40 µm	14
**$Li_4Ti_5O_{12}$ (1C = 175 mA g^{-1})**						
15	Flexible and self-standing electrodes *via* vacuum filtration	LTO: MWCNTs= 85: 15	5.25 mg cm^{-2} (LTO: 4.46 mg cm^{-2})	Particle size: 2 µm	25 µm	15
16	Mesoporous spinel $Li_4Ti_5O_{12}$ nanosheets	AM: AB: PTFE = 80: 10: 10	10.6 mg cm^{-2} (AM: 8.5 mg cm^{-2})	Mesoporous nanosheets: 150−200 nm	30 µm	16
17	Rutile-TiO_2 nanocoated $Li_4Ti_5O_{12}$ nanosheets	AM: Super P: PVDF = 80: 10: 10	12.5 mg cm^{-2} (AM: 10 mg cm^{-2})	Nanosheets thickness: 30−60 nm (AM: 1.5 g cm^{-3})	≈ 100 µm (AM: 70 µm)	17
**Graphite (1C = 372 mA g^{-1})**						
18	Magnetically aligned graphite electrodes	Large graphite flakes: Small flakes: PVP: Super P= 15.4: 3.8: 2.4: 2.3	6.3 mg cm^{-2} (Graphite: 5 mg cm^{-2})	Graphite size: Large flake ≤ 44 µm small flakes: 7−10 µm	≈ 110 µm	18
19	Coating of graphite anode with coal tar pitch as an effective precursor	AM: SBR: CMC = 90: 5: 5	6 mg cm^{-2} (AM: 5.4 mg cm^{-2})	particle size 10 µm Active material: 1.5 g cm^{-3}	40 µm	19

Supplementary Table 3. Literature details for Cohort IB (data sets with varying electrode thickness)

Row No.	Electrode	Electrode details	Electrolyte details	Separator thickness	Ref.
**Theoretical density:<br>NMC-4.77 g cm^{-3}<br>LFP-3.6 g cm^{-3}<br>Graphite-2.25 g cm^{-3}**					
1	Slurry-casted NMC111/LFP electrode	NMC111<br>Thickness: 24−104 µm<br>Density: 2.7 g cm^{-3}<br>Porosity: 44%<br>LFP<br>Thickness: 10−108 µm<br>Density: 1.8 g cm^{-3}<br>Porosity: 50%	1 M $LiPF_6$ in EC/DEC=1:1<br>$\sigma_{BL} \approx 0.8$ S m^{-1}	Unknown	[2]
2	Slurry-casted NMC622 or graphite electrodes with different areal capacity	NMC622<br>Thickness: 48−154 µm<br>Porosity: 32−37%<br>Graphite<br>Thickness: 58−182 µm<br>Porosity: 37−40%	1 M $LiPF_6$ in EC/EMC (3:7 weight ratio) + additional 2 wt.% vinylene carbonate (BASF)<br>$\sigma_{BL} \approx 0.5$ S m^{-1}	Unknown	[20]
**Nb_2O_5/Graphene composite-3.85 g cm^{-3}**					
3	Three-dimensional holey-graphene/niobia composites	Electrode porosity: ≈ 60%.	1 M $LiPF_6$ in EC/DMC (1:1 volume ratio, BASF)<br>$\sigma_{BL} \approx 1.16$ S m^{-1}	Unknown	[21]
**LFP-3.6 g cm^{-3}**					
4	$LiFePO_4$ cathodes with different electrode parameters	Density: 2.2 *vs.* 1.55 g cm^{-3}.<br>Porosity: 40% *vs.* 57%.	0.7, 1.0, and 1.3 M $LiPF_6$ in EC/DEC or EC/DMC (3:7 by volume ratio)<br>σ_{BL}:<br>1 M $LiPF_6$ in EC/DEC (3:7): 0.724 S m^{-1}<br>1 M $LiPF_6$ in EC/DMC (3:7): 1.872 S m^{-1}	Unknown	[22]
**GaS - 3.86 g cm^{-3}**					

5	Liquid-phase exfoliated 2D gallium chalcogenide nanosheet/CNT composites	Thickness: 5−29 µm<br>Density: 0.44−0.6 g cm^{-3}<br>Porosity: 84−89%.	1 M $LiClO_4$ in EC/DMC (1:1 volume ratio)<br>$\sigma_{BL} \approx 0.5$ S m^{-1}	50 µm	[23]
**Data collected by us**<br>**Si-2.33 g cm^{-3}**<br>**LTO-3.42 g cm^{-3}**<br>**NMC-4.77 g cm^{-3}**					
6	LTO/CNT	Thickness: 5−100 µm<br>Density: 0.5 g cm^{-3}<br>Porosity: 85%.	1 M $LiPF_6$ in EC/DEC/FEC, (3:6:1 volume ratio, BASF) with 2 wt% VC<br>$\sigma_{BL} \approx 0.5$−1.0 S m^{-1}	25 µm	This work
7	NMC/CNT	Thickness: 115−540 µm<br>Density: 1.75−2.13 g cm^{-3}<br>Porosity: 55−63 %.	Half Cell:<br>1 M $LiPF_6$ in EC/DEC/FEC (3:6:1 volume ratio, BASF) with 2 wt% VC<br>$\sigma_{BL} \approx 0.5$−1.0 S m^{-1}	25 µm	This work
8	SiGr/CNT	Thickness: 30−300 µm<br>Density: 0.4 g cm^{-3}<br>Porosity: 83%.	1 M $LiPF_6$ in EC/DEC/FEC (3:6:1 volume ratio, BASF) with 2 wt% VC<br>$\sigma_{BL} \approx 0.5$−1.0 S m^{-1}	25 µm	This work
9	µSi/CNT	Thickness: 30−170 µm<br>Density: 0.43−0.64 g cm^{-3}<br>Porosity: 73−82%.	1 M $LiPF_6$ in EC/DEC/FEC (3:6:1 volume ratio, BASF) with 2 wt% VC<br>$\sigma_{BL} \approx 0.5$−1.0 S m^{-1}	25 µm	This work

Cohort Ⅱ: Sodium ion batteries

Supplementary Table 4. Literature details for Cohort II (Na-ion batteries)

Row No.	Electrode	Electrode compositio n	Mass loading (M_{Total}/A and M_{AM}/A)	Particle size, morphology or density	Electrode thickness	Ref.
**Cathode**						
**$Na_{0.44}MnO_2$ ($Na_4Mn_9O_{18}$) (1C = 120 mA g^{-1})**						
1	$Na_{0.44}MnO_2$ nanoplate cathode	AM: Super C65: PVDF = 80: 10: 10	2 mg cm^{-2} (AM: 1.6 mg cm^{-2})	Particle size: 6 µm in length and 2 µm in width	≈ 10−20 µm	24
**$Na_2FeP_2O_7$ (1C = 97 mA g^{-1}) Theoretical density: 3.15 g cm^{-3}**						
2	$Na_2FeP_2O_7$ nanoparticles on porous carbon cloth	AM: Super P: PVDF = 70: 15: 15	1.4−2.1 mg cm^{-2} (NMC: 1−1.5 mg cm^{-2})	Particle size: 20 − 50 nm Particles embedded in carbon	≈ 10 µm	25
**Na_2FePO_4F (1C = 124 mA g^{-1}) Theoretical density: 3.32 g cm^{-3}**						
3	Na_2FePO_4F nanoparticles by enhancing surface carbon functionality	AM: Super P: PVDF = 70: 20: 10 (AM mixed by Na_2FePO_4F : GO = 80/20)	1−2 mg cm^{-2} (AM: 0.7−1.4 mg cm^{-2})	Particle size: 25 nm	< 10 µm (doctor-blade 10 µm)	26
**$Na_2VTi(PO_4)_3$ (1C = 147 mA g^{-1})**						
4	$Na_2VTi(PO_4)_3$ as ionic conductor structured electrode	AM: Super P: PVDF = 70: 20: 10	1.75−2.2 mg cm^{-2} (AM: 1.225−1.54 mg cm^{-2})	Particle size: ≈ 200 nm	≈ 15−20 µm	27
**$Na_3V_2(PO_4)_3$ (1C = 110 mA g^{-1}) Theoretical density: 3.21 g cm^{-3}**						
5	Carbon-coated $Na_3V_2(PO_4)_3$ embedded in porous carbon matrix	AM: Super P: PVDF = 70: 20: 10	1.43 mg cm^{-2} (AM: 1 mg cm^{-2})	Particle size: 60 nm	≈ 20 µm	28
6	Hierarchical carbon framework wrapped $Na_3V_2(PO_4)_3$ as a cathode	AM: AB: PVDF = 70: 20: 10	1.43 mg cm^{-2} (AM: 1 mg cm^{-2})	Particle size: 100−500 nm	≈ 15 µm	29

7	Layer-by-Layer $Na_3V_2(PO_4)_3$ embedded in rGO	AM: CB: PVDF = 70: 20: 10	1.5−2.0 mg cm^{-2} (AM: 1−1.4 mg cm^{-2})	Particle size: 200 nm	≈ 15−20 µm	30
**$NaCrO_2$ (1C = 110 mA g^{-1})** **Theoretical density: 4.36 g cm^{-3}**						
8	Layered O_3-type carbon-coated $NaCrO_2$ cathode for Na-ion battery (Yu *et al.*)	AM: KS-6: Super P: PVDF = 85: 3.75: 3.75: 7.5 All samples with x wt% carbon coating (0<x<16.6%)+7.5% (KS-6+superP)	4.1 mg cm^{-2} (AM: 3.5 mg cm^{-2})	Particle size: ≈ 500 nm	≈ 20 µm	31
**$FePO_4$ (1C = 178 mA g^{-1})** **Theoretical density 2.87 g cm^{-3}**						
9	Mesoporous $FePO_4$ nanospheres as cathode	AM: KB: PVDF = 70: 20: 10	2 mg cm^{-2} (AM: 1.4 mg cm^{-2})	Particle size: 100 nm	≈ 30 µm	32
**Anode**						
**TiO_2 (1C = 335 mA g^{-1})** **Theoretical density: 4.23 g cm^{-3}**						
10	Carbon-coated anatase TiO_2 anode	TiO_2: Carbon coating: Super C65: PVDF = 65:12:13:10	1.85−2.31 mg cm^{-2} (TiO_2: 1.2−1.5 mg cm^{-2})	Particle size: 11 nm	< 120 µm (doctor-blade) ≈ 20−30 µm	33
		TiO_2: Carbon coating: Super C65: PVDF = 65:9:16:10	1.85−2.77 mg cm^{-2} (TiO_2: 1.2−1.8 mg cm^{-2})	Particle size: 40 nm		
		TiO_2: Carbon coating: Super C65: PVDF = 65:0:25:10	1.85 mg cm^{-2} (TiO_2: 1.2 mg cm^{-2})	Particle size: 15 nm		

**Carbon (1C = 372 mA g^{-1})**<br>**Theoretical density: 2.26 g cm^{-3}**						
11	Hard carbon anode *via* graphene oxide as dehydration agent	AM: PVDF = 93: 7	2.7 mg cm^{-2}<br>(AM: 2.5 mg cm^{-2})	Without GO particle size: 30−80 µm	40 µm	34
				With GO particle size: 80−110 µm		
**Graphite (1C = 372 mA g^{-1})**<br>**Theoretical density: 2.28 g cm^{-3}**						
12	Expanded graphite (EG) as anode	EG: Na alginate<br>= 85: 15	0.6 mg cm^{-2}<br>(EG: 0.5 mg cm^{-2})	(Unknown)	≈ 10 µm	35
13	Natural graphite using ether-based electrolytes	AM: CB: PVDF<br>= 70: 20: 10	4.3 mg cm^{-2}<br>(AM: 3 mg cm^{-2})	Particle size<br>100 µm	45 µm	36
**Sb (1C = 660 mA g^{-1})**<br>**Theoretical density: 6.68 g cm^{-3}**						
14	Monodispersed Sb nanocrystals for Li-/Na-ion battery anodes	AM: CB: CMC<br>= 64: 21: 15	1 mg cm^{-2}<br>(AM: 0.64 mg cm^{-2})	Particle size: ≤ 74 µm	10−15 µm	37
				Particle size: 10 nm		
				Particle size: 20 nm		
15	Rod-like Sb–C composite as anodes for Li-/ Na-ion batteries	AM: CB: PVDF<br>= 70: 20: 10	3 mg cm^{-2}<br>(AM: 2 mg cm^{-2})	Secondary micro-rods: 6 µm in length, 2 µm in diameter<br>Primary nanoparticles size: 200 nm	≈ 40 µm	38

Cohort Ш: Varying conductive additive Mf

Supplementary Table 5. Literature details for Cohort Ш

Row No.	Electrode	Electrode composition	Mass loading (M_{Total}/A and M_{AM}/A)	Particle size, morphology or density	Electrode thickness	Ref.
**$Li_4Ti_5O_{12}$ (1C = 175 mA g^{-1})**						
1	LTO/SWCNT composites (Coelho *et al.*)	Different mass fractions of CNTs	AM: 0.2 mg cm^{-2}	Particle size: 174 nm	1.1 μm	39
**MoS_2**						
2	MoS_2 intercalated p-Ti_3C_2 anode (Zheng *et al.*)	AM: AB: PVDF = 80: 10: 10	Unknown	Unknown	Unknown	40
**MWCNT (1C = 400 mA g^{-1})**						
3	CuNW-MWCNT composite anodes (Yin *et al.*)	CuNW:MWCNT = 1:1, 3:1, 5:1, and 7:1, w/w	CNT: 1.46 mg cm^{-2}	CuNW with D ≈ 100 nm CNT with D = 10 nm	61−126 μm	41
**$NaTi_2(PO_4)_3$ (1C = 133 mA g^{-1})**						
4	CNT decorated $NaTi_2(PO_4)_3$/C nanocomposite for Na-ion battery anode (Wang *et al.*)	AM: AB: PVDF = 80: 10: 10	2 mg cm^{-2} (AM: 1.6 mg cm^{-2})	Particle size: < 100 nm	Unknown	42
**SnS_2/MXene**						
5	2D MXene/SnS_2 composites for Na-ion battery anode (Wu *et al.*)	AM: AB: CMC = 80: 10: 10 (AM: MX/SnS_2 = 5/1, 10/1 and 2/1)	Unknown	SnS_2 size: 20–25 nm	Unknown	43
**GaS (1C = 1107 mA g^{-1})**						
6	2D Gallium Chalcogenide Nanosheet/CNT composites (Zhang *et al.*)	Different mass fractions of CNTs (SWCNT ：5%, 10%, 20%, 30%, and 50%)	2 mg cm^{-2}	GaS NS size: 338 nm	5 μm	23

**$NaCrO_2$ (1C = 110 mA g^{-1})<br>Theoretical density: 4.36 g cm^{-3}**						
7	Layered O_3-type carbon-coated $NaCrO_2$ cathode for Na-ion battery (Yu *et al.*)	AM: KS-6: Super P: PVDF = 85: 3.75: 3.75: 7.5 All samples with x wt% carbon coating (0<x<16.6%)+7.5 % (KS-6+superP)	4.1 mg cm^{-2} (AM: 3.5 mg cm^{-2})	particle size: ≈ 500 nm	≈ 20 µm	[31]
**$Li_4Ti_5O_{12}$ (1C = 175 mA g^{-1})**						
8	$Li_4Ti_5O_{12}$/Graphene anodes for Li-ion batteries	PVDF=10 wt% (fixed) Graphene contents varying from 0 to 10 wt%	Unknown	LTO rods: 1–2 µm length GNS thickness: ≈ 4.5 nm GNS diameter: ≈ 46 mm	Unknown	[44]
**$LiCoO_2$ (1C = 140 mA g^{-1})**						
9	$LiCoO_2$/Graphene/Super P based Li-ion batteries	Different contents of graphene nanosheet (GN) together with Super-P (SP). No information about binder	Electrode: 10 mg cm^{-2}	LCO particle size: 5−10 µm	≈ 50 µm	[45]
**$LiFePO_4$ (1C = 170 mA g^{-1})**						
10	$LiFePO_4$/Graphene cathode for Li-ion batteries	1% GN + 9% SP. No information about binder	Unknown	$LiFePO_4$ microparticle: 400–600 nm Single-layer + multi-layer GNS (3–15 layer) GN1: 1–2 µm; GN2: 3–5 µm; GN3: 10–15 µm;	Unknown	[46]
**NMC111 (1C = 155 mA g^{-1})**						
11	NMC/graphite/CB cathode for Li-ion batteries	AM: CB: PVDF = 92: 4: 4	Unknown	NMC111: 8.9 µm. Carbon black (Super C65): 35 nm.	Unknown	[47]

Supplementary Table 6. Details of electrode parameters in Figure 6.

Parameters		Values	Details	Ref.
σ_E	Out-of-plane electrode conductivity	10 S m^{-1}	High enough to remove resistance limitation in the electrode	This work
σ_{BL}	Bulk liquid conductivity of electrolyte	0.5 S m^{-1}	Midpoint of the literature values (σ_{BL} = 0.1−1 S m^{-1})	[48]
P_E	Porosity of electrode	0.5 (50 %)	Typical value from references (P_E = 0.3−0.6)	[2,20,22]
P_S	Porosity of separator	0.4 (40 %)	Typical porosity of the standard Celgard separator (P_S = 0.35−0.45)	[49]
L_E	Battery electrode thickness	120 μm	Typical value for electrode in commercial battery. (L_E = 100−150 μm)	[20]
L_S	Battery separator thickness	25 μm	Thickness of the standard Celgard separator	[52]
L_{AM}	Active material thickness	100 nm	Equivalent to particle diameter of 600 nm	[50]
$C_{V,eff}$	Effective volumetric capacitance associated with battery electrode	2×10^4 F cm^{-3}	Roughly in midpoint of the range found from our model ($C_{V,eff}$ = 10^3–10^5 F cm^{-3})	This work
D_{BL}	Bulk liquid diffusion coefficient of electrolyte	3×10^{-10} m^2 s^{-1}	Midpoint of the literature values (D_{BL} = $1-5\times10^{-10}$ m^2 s^{-1})	[51–53]
D_{AM}	Li ion diffusion coefficient in the solid active material	10^{-16} m^2 s^{-1}	Typical value from reference.	[54]
t_c	Characteristic time associated with electrochemical reaction at the electrode/electrolyte interface	25 s	Within the range of literature values	[50]

Supplementary Notes

Supplementary Note 1. Deriving the equation for characteristic time

This parameter is a measure of R_T so is likely to be controlled by physical properties of the electrode. This data outlined above implies that τ has both resistance and diffusive contributions. In addition, we must include the effects of the kinetics of the electrochemical reaction at the electrode/electrolyte interface. This can be done *via* the characteristic time associated with the reaction, t_c, which is primarily controlled by the exchange current density *via* the Butler-Volmer equation.[50] This characteristic time can range from ~0.1 to >100 s, depending on the circumstances.[50]

Then, τ is the sum of the three contributing factors:

$$\tau = \tau_{Electrical} + \tau_{Diffusive} + t_c \tag{5A}$$

It is likely that that the diffusive component is just the sum of diffusion times associated with *cation* transport in the electrolyte, both within the separator (coefficient D_S) and the electrolyte-filled pores within the electrode (coefficient D_P) as well as in the solid active material (coefficient D_{AM}).[50] These times can be estimated using $L = \sqrt{Dt}$ such that

$$\tau_{Diffusive} = \frac{L_E^2}{D_P} + \frac{L_S^2}{D_S} + \frac{L_{AM}^2}{D_{AM}} \tag{5B}$$

where L_E, L_S and L_{AM} are the electrode thickness, separator thickness and the length scale associated with active material particles respectively. We note that the value of L_{AM} depends on material geometry. For a thin film of active material, L_{AM} is the film thickness while for a quasi-spherical particle of radius r,[50] $L_{AM}=r/3$.

For the electrical contribution, we note that every battery electrode has an associated *capacitance*[55] which limits the rate at which the electrode can be charged/discharged. This effective capacitance, C_{eff}, will be dominated by charge storage but may also have contributions due to surface or polarisation effects.[55] Then, we propose $\tau_{Electrical}$ to be the RC time constant associated with the circuit. The total resistance related to the charge/discharge process is the sum of the resistances due to out-of-plane electron transport in the electrode material ($R_{E,E}$), as well as ion transport, both in the electrolyte-filled pores of the electrode ($R_{I,P}$) and in the separator respectively ($R_{I,S}$). Then, the RC contribution to τ is given by

$$\tau_{Electrical} = C_{eff}(R_{E,E} + R_{I,P} + R_{I,S}) \quad (5C)$$

The overall characteristic time associated with charge/discharge is then the sum of capacitive and diffusive components and t_c:

$$\tau = C_{eff}(R_{E,E} + R_{I,P} + R_{I,S}) + \frac{L_E^2}{D_P} + \frac{L_S^2}{D_S} + \frac{L_{AM}^2}{D_{AM}} + t_c \quad (5D)$$

In porous structures, only a fraction of the total volume is available for transport, thus reducing the diffusion coefficient relative to that in the bulk liquid (D_{BL}). This is dealt with *via* the Bruggeman equation which says: $D_{porous} = D_{BL}P/\tau$ where P is porosity and tau is tortuosity. This is usually simplified *via* the approximation $\tau = P^{-1/2}$ so $D_{Porous} = D_{BL}P^{3/2}$ We apply this approach to relate the diffusion coefficients of ions in the separator and porous electrode (D_S and D_P) to that in bulk liquid: $D_P = D_{BL}P_E^{3/2}$ and $D_S = D_{Bulk}P_S^{3/2}$. Here, P_E and P_S represent the porosity of the electrode and separator respectively.

We can generalise this approach to apply to ionic conductivity because the Nearnst-Einstein predicts a linear relationship between ionic conductivity and the diffusion coefficient. Then, we can write the resistances as $R_{E,M} = \frac{L_E/2}{\sigma_E A}$, $R_{I,P} = \frac{L_E/2}{\sigma_{BL}P_E^{3/2}A}$ and $R_{I,S} = \frac{L_S}{\sigma_{BL}P_S^{3/2}A}$, where σ_{BL} is the conductivity of the bulk liquid electrolyte and A is the area of both the electrode and separator. The factor of 2 comes from that fact that, on average, ions from the electrolyte and electrons from the current collector meet at the midpoint of the electrode. Combining these expressions then gives

$$\tau = \frac{C_{eff}}{A}\left[\frac{L_E}{2\sigma_E} + \frac{L_E}{2\sigma_{BL}P_E^{3/2}} + \frac{L_S}{\sigma_{BL}P_S^{3/2}}\right] + \frac{L_E^2}{D_{BL}P_E^{3/2}} + \frac{L_L^2}{D_{BL}P_S^{3/2}} + \frac{L_S^2}{D_S} + t_c$$

The effective capacitance of the electrode probably scales with electrode thickness (constant volumetric capacitance, $C_{eff}/AL_E = C_{V,eff}$) so

$$\tau = C_{V,eff}\left[\frac{L_E^2}{2\sigma_E} + \frac{L_E^2}{2\sigma_{BL}P_E^{3/2}} + \frac{L_E L_S}{\sigma_{BL}P_S^{3/2}}\right] + \frac{L_E^2}{D_{BL}P_E^{3/2}} + \frac{L_S^2}{D_{BL}P_S^{3/2}} + \frac{L_{AM}^2}{D_{AM}} + t_c$$

Which can be rearranged as

$$\tau = L_E^2\left[\frac{C_{V,eff}}{2\sigma_E} + \frac{C_{V,eff}}{2\sigma_{BL}P_E^{3/2}} + \frac{1}{D_{BL}P_E^{3/2}}\right] + L_E\left[\frac{L_S C_{V,eff}}{\sigma_{BL}P_S^{3/2}}\right] + \left[\frac{L_S^2}{D_{BL}P_S^{3/2}} + \frac{L_{AM}^2}{D_{AM}}\right] + t_c$$

Or alternatively, keeping electrical and diffusive terms together and writing as Θ:

$$\Theta^{-1} = \frac{\tau}{L_E^2} = C_{V,eff}\left[\frac{1}{2\sigma_E} + \frac{1}{2\sigma_{BL}P_E^{3/2}} + \frac{L_S / L_E}{\sigma_{BL}P_S^{3/2}}\right] + \left[\frac{1}{D_{BL}P_E^{3/2}} + \frac{L_S^2 / L_E^2}{D_{BL}P_S^{3/2}} + \frac{L_{AM}^2 / L_E^2}{D_{AM}}\right] + \frac{t_c}{L_E^2}$$

Supplementary Note 2. Calculating Percolation Parameters

The data in figure 3E is described by

$$\tau / L_E^2 = \frac{C_{V,eff} / 2}{\sigma_M + \sigma_0 (M_f)^s} + \beta_1$$

For fitting purposes, this can be written as

$$\tau / L_E^2 = \frac{(C_{eff} / V) / 2\sigma_M}{1 + (\sigma_0 / \sigma_M)(M_f)^s} + \beta_1$$

From the fits in figure 3E and figure 5A, we obtain the parameters $(C_{eff} / V) / 2\sigma_M$, σ_0 / σ_M, s and β_1 as shown in table 1. The mean volumetric capacity, Q_V, can be found by multiplying Q_M by the electrode density which can usually be extracted/estimated from data in papers. The effective volumetric capacitance was found from $C_{V,eff} = 28 \times \rho_E C_M$ (figure 4F). This parameter can be combined with the fit parameters to yield σ_M and σ_0 (Supplementary Table 1).

The percolation exponents found ranged from 1.4 to 3.5 in good agreement with the fact that in the literature, such exponents usually fall in the range 1.4<s<4.[56] The σ_M parameter is a measure of the out-of-plane conductivity of the electrode in the absence of conductive additive. This parameter should be quite small, usually <<1 S m^{-1} as is found below. We note that the Yu paper has a higher value of σ_M =0.18 S m^{-1}. This is as expected because the active particles making up the electrode were coated with a conductive carbon coating yielding some conductivity even in the absence of additional filler. The parameter σ_0 is roughly a measure of the out-of-plane conductivity of a network containing only the filler particles which we expect to be ~1000x lower than the in plane conductivity. σ_0 can be quite high, perhaps reaching ~10^6 S m^{-1} for graphite.[57] Thus, the value obtained for Yu *et al* appears plausible while the value obtained from B Zhang's paper is reasonably consistent with their in-plane conductivity which implies a value of σ_0 somewhat larger than 10^6 S m^{-1}. Notable, the values of σ_0 found for J Zhang and J Coelho's work are very low. These papers describe electrodes with carbon nanotubes added to increase conductivity. One might expect NTs to give very good conductivity enhancements and so large values of σ_0. However, in composites prepared by liquid processing, nanotube tend to become aligned in-plane. This can give very low out-of-

plane conductivities. For example, we previously reported and out-of-plane σ_0 value of 4 S m^{-1} for a composite of single walled nanotubes mixed with $Co(OH)_2$ nanosheets.[58] This data implies that nanotubes may not be as good at enhancing rate as may have been thought.

Supplementary Note 3. Main text Fig 5 Calculations

Figure 5B

Bauer *et al*: Characteristic time *versus* porosity.

Equation: $\frac{\tau}{L_E^2}=\left[\frac{C_{V,eff}}{2\sigma_{BL}}+\frac{1}{D_{BL}}\right]P_E^{-3/2}+\beta_2$

Fit equation: $\frac{\tau}{L_E^2}$=1.27e10*P^ (-3/2)+9.8e8

From the fit, the square-bracketed term=1.27×10^{10} s m^{-2}. Personal communication from the author showed the electrodes to have L_E=62 and 77 μm for the low- and high-P samples respectively. The volumetric capacities (Q_V) were ~3×10^8 mAh m^{-3}. Using the relationship in figure 4F ($C_{V,eff}=28\times Q_V$) gives $C_{V,eff}$ =8.4×10^9 F m^{-3}. Then approximating D_{BL} ≈3×10^{-10} m^2 s^{-1} yields σ_{BL}=0.45 S m^{-1}. As typical electrolytes used in LiBs (c=1 M) typically have σ_{BL}~0.5–1 S m^{-1}, this is as expected.

Figure 5C

Yu *et al*: Characteristic time *versus* electrolyte concentration

Equation: $\frac{\tau}{L_E^2}=\left[\frac{C_{V,eff}}{2\sigma_E}+\frac{1}{D_{BL}P_E^{3/2}}\right]+\frac{t^+RT}{F^2D_{BL}c}\left[\frac{C_{V,eff}}{2P_E^{3/2}}+\frac{L_S}{L_E}\frac{C_{V,eff}}{P_S^{3/2}}\right]$

Fit equation; $\frac{\tau}{L_E^2}$=1.6e10+9.1e13/c (c in mol m^{-3})

The volumetric capacities were ~3.6×10^8 mAh m^{-3}. Using the relationship in figure 4F ($C_{V,eff}=28\times Q_V$) gives $C_{V,eff}$ =10.1×10^9 F m^{-3}.

Then approximating t^+≈1, $P_S\approx0.4$, P_E~0.4 (estimated from paper=1-(2.2 g cc^{-1})/(3.6 g cc^{-1})) and taking D_{BL}≈10^{-10} m^2 s^{-1} and using the slope yields L_S/L_E=0.35. Assuming, L_S=25 μm, yields L_E=71 μm, close to the quoted value of 65 μm.

Figure 5D

This work: Characteristic time *versus* electrolyte (separator) thickness.

Equation: $\frac{\tau}{L_E^2} = L_S \left[\frac{C_{V,eff}}{L_E \sigma_{BL} P_S^{3/2}} \right] + \beta_3$

Fit: 2.04e10+2.71e14*L_S

The volumetric capacities were ~3.3×10^8 mAh m^{-3}. Using the relationship in figure 4F ($C_{V,eff} = 28 \times Q_V$) gives $C_{V,eff}$ =9.2×10^9 F m^{-3}.

Then approximating D_{BL} ≈3×10^{-10} m^2 s^{-1} and P_S~0.4 and taking the measured value of L_E=245 μm gives σ_{BL} ~0.6 S m^{-1}.

Figure 5E

Ye *et al*: characteristic time versus thickness of thin active layer in porous current collector (large pores)

Equation: $\tau = \frac{L_{AM}^2}{D_{AM}} + \beta_4$

From the fit $1/D_{AM}$=3.02×10^{18} s m^{-2} yielding D_{AM}=3.3×10^{-19} m^2 s^{-1}.

Supplementary Methods

Electrode fabrication and electrochemical characterization performed in this work

As shown in figure 4 and 5D (main text), some electrodes were prepared and tested in this work. All such electrodes were prepared by a conventional slurry casting method using a CNT solution (Tuball, OCSiAl, 0.2 wt% SWCNT in water with ~0.2 wt% PVP as a surfactant) with various battery active materials (AM). Here we employed nano-LTO (~200 nm, Aldrich), micro-sized silicon (1–3 µm, US Research Nanomaterials), and graphene wrapped silicon (Si/Gr, Nano GCA-2000, Angstron Materials) for anodes, and NMC (NMC811, ~10 µm, Li-fun Tech) for cathodes, respectively. The AM powers were directly mixed with the CNT solution and ground by a mortar and pestle to obtain a uniform slurry. The CNT contents and electrode compositions were controlled by changing the mixing ratio between CNT solution and AM power. For instance, 1 g of AM powder was mixed with 2.5 ml of CNT solution to fabricate 0.5 wt% CNT-AM electrode. Then the slurry was cast onto either Al or Cu foil using a doctor blade. The electrode thickness was also controlled by the slurry coating thickness with changing the height of the doctor blade. Then the slurry cast electrodes were slowly dried at 40 °C for 2 hours and followed by vacuum drying at 100 °C for 12 hours to remove residual water.

The electrochemical properties of the electrodes were measured in 2032-type coin cells (MTI Corp.) with a half-cell configuration. All coin cells were assembled in an Ar-filled glovebox (UNIlab Pro, Mbraun). The dried electrodes were cut into 12 mm diameter discs (area = 1.13 cm^2) and paired with Li metal discs (diameter= 16 mm). Celgard 2032 (thickness = 25 µm) was used as a separator. To investigate the effect of separator thickness, we also varied the number of stacked separators (1−3 stacks, thickness varying from 25−75 µm). The electrolyte was 1−1.2 M $LiPF_6$ dissolved in EC/DEC/FEC (3:6:1 in v/v/v, BASF) with 2 wt% vinylene carbonate. The rate-capabilities were evaluated by galvanostatic charge-discharge (GCD) test at a potentiostat (VMP3, Biologic). The voltage range was 0.005−1.2 V, 1−2.5 V and 3−4.3 V for Si, LTO, and NMC electrodes, respectively.

Capacity-rate analysis in this work

Note on fitting

All the fitting analysis has been performed by Origin software (here we used Origin version 2015-2018) using “Nonlinear Curve Fit” function, according to the model equation.

Although it seems obvious that the best way to fit data is to plot *Q/M* v *R* and use $\frac{Q}{M} = Q_M \left[1 - (R\tau)^n \left(1 - e^{-(R\tau)^{-n}} \right) \right]$ to fit, this is not the case. The reason is that in some cases, the *Q*/*M* data can span a wide range. Then the least squares fitting routine is biased toward the larger values of *Q/M* leading to poor fits at high *R*.

To avoid this, we plotted the data as log(*Q/M*) *vs*. *R* and fitted using

$$\log(Q / M) = \log \left\{ Q_M \left[1 - (R\tau)^n \left(1 - e^{-(R\tau)^{-n}} \right) \right] \right\}$$

The resultant smaller range of y-values results in much better fitting.

Supplementary References

1. Yang, C.F., Zhang, X.S., Huang, M.Y., Huang, J.J. & Fang, Z.B. Preparation and Rate Capability of Carbon Coated LiNi1/3Co1/3Mn1/3O2 as Cathode Material in Lithium Ion Batteries. *Acs Appl Mater Inter* **9**, 12408-12415 (2017).
2. Zheng, H.H., Li, J., Song, X.Y., Liu, G. & Battaglia, V.S. A comprehensive understanding of electrode thickness effects on the electrochemical performances of Li-ion battery cathodes. *Electrochim Acta* **71**, 258-265 (2012).
3. Singh, M., Kaiser, J. & Hahn, H. A systematic study of thick electrodes for high energy lithium ion batteries. *J Electroanal Chem* **782**, 245-249 (2016).
4. Kim, J.M., Park, C.H., Wu, Q.L. & Lee, S.Y. 1D Building Blocks-Intermingled Heteronanomats as a Platform Architecture For High-Performance Ultrahigh-Capacity Lithium-Ion Battery Cathodes. *Adv Energy Mater* **6**, 1501594 (2016).
5. Gaikwad, A.M. *et al.* A High Areal Capacity Flexible Lithium-Ion Battery with a Strain-Compliant Design. *Adv Energy Mater* **5,** 1401389 (2015).
6. Lai, W. *et al.* Ultrahigh-Energy-Density Microbatteries Enabled by New Electrode Architecture and Micropackaging Design. *Adv Mater* **22**, E139-E144 (2010).
7. Cho, S.J. *et al.* Hetero-Nanonet Rechargeable Paper Batteries: Toward Ultrahigh Energy Density and Origami Foldability. *Adv Funct Mater* **25**, 6029-6040 (2015).
8. Ren, W.J. *et al.* Soft-contact conductive carbon enabling depolarization of LiFePO4 cathodes to enhance both capacity and rate performances of lithium ion batteries. *J Power Sources* **331**, 232-239 (2016).
9. Lou, X.M. & Zhang, Y.X. Synthesis of LiFePO4/C cathode materials with both high-rate capability and high tap density for lithium-ion batteries. *J Mater Chem* **21**, 4156-4160 (2011).
10. Yang, G.F., Song, K.Y. & Joo, S.K. Ultra-thick Li-ion battery electrodes using different cell size of metal foam current collectors. *Rsc Adv* **5**, 16702-16706 (2015).
11. Shao, D., Zhong, H.X. & Zhang, L.Z. Water-Soluble Conductive Composite Binder Containing PEDOT: PSS as Conduction Promoting Agent for Si Anode of Lithium-Ion Batteries. *Chemelectrochem* **1**, 1679-1687 (2014).
12. Leveau, L. *et al.* Silicon nano-trees as high areal capacity anodes for lithium-ion batteries. *J Power Sources* **316**, 1-7 (2016).
13. Zhou, M. *et al.* High-Performance Silicon Battery Anodes Enabled by Engineering Graphene Assemblies. *Nano Lett* **15**, 6222-6228 (2015).
14. Salvatierra, R.V. *et al.* Silicon Nanowires and Lithium Cobalt Oxide Nanowires in Graphene Nanoribbon Papers for Full Lithium Ion Battery. *Adv Energy Mater* **6,** 1600918 (2016).
15. Bourgeois, J.P., Vlad, A., Melinte, S. & Gohy, J.F. Design of Flexible and Self-Standing Electrodes for Li-Ion Batteries. *Chinese J Chem* **35**, 41-47 (2017).
16. Ge, H. *et al.* Unique mesoporous spinel Li4Ti5O12 nanosheets as anode materials for lithium-ion batteries. *Journal of Power Sources* **297**, 436-441 (2015).
17. Wang, Y.Q. *et al.* Rutile-TiO2 Nanocoating for a High-Rate Li4Ti5O12 Anode of a Lithium-Ion Battery. *J Am Chem Soc* **134**, 7874-7879 (2012).
18. Billaud, J., Bouville, F., Magrini, T., Villevieille, C. & Studart, A.R. Magnetically aligned graphite electrodes for high-rate performance Li-ion batteries. *Nat Energy* **1,** 16097 (2016).
19. Han, Y.J. *et al.* Coating of graphite anode with coal tar pitch as an effective precursor for enhancing the rate performance in Li-ion batteries: Effects of composition and softening points of coal tar pitch. *Carbon* **94**, 432-438 (2015).

20. Gallagher, K.G. *et al.* Optimizing Areal Capacities through Understanding the Limitations of Lithium-Ion Electrodes. *J Electrochem Soc* **163**, A138-A149 (2016).

21. Sun, H.T. *et al.* Three-dimensional holey-graphene/niobia composite architectures for ultrahigh-rate energy storage. *Science* **356**, 599-604 (2017).

22. Yu, D.Y.W., Donoue, K., Inoue, T., Fujimoto, M. & Fujitani, S. Effect of electrode parameters on LiFePO4 cathodes. *J Electrochem Soc* **153**, A835-A839 (2006).

23. Zhang, C.F. *et al.* Enabling Flexible Heterostructures for Li-Ion Battery Anodes Based on Nanotube and Liquid-Phase Exfoliated 2D Gallium Chalcogenide Nanosheet Colloidal Solutions. *Small* **13,** 1701677 (2017).

24. He, X. *et al.* Durable high-rate capability Na0.44MnO2 cathode material for sodium-ion batteries. *Nano Energy* **27**, 602-610 (2016).

25. Song, H.J., Kim, D.S., Kim, J.C., Hong, S.H. & Kim, D.W. An approach to flexible Na-ion batteries with exceptional rate capability and long lifespan using Na2FeP2O7 nanoparticles on porous carbon cloth. *Journal of Materials Chemistry A* **5**, 5502-5510 (2017).

26. Ko, J.S. *et al.* High-rate capability of Na2FePO4F nanoparticles by enhancing surface carbon functionality for Na-ion batteries. *Journal of Materials Chemistry A* **5**, 18707-18715 (2017).

27. Wang, D.X. *et al.* Sodium vanadium titanium phosphate electrode for symmetric sodium-ion batteries with high power and long lifespan. *Nature Communications* **8,** 15888 (2017).

28. Zhu, C.B., Song, K.P., van Aken, P.A., Maier, J. & Yu, Y. Carbon-Coated Na3V2(PO4)(3) Embedded in Porous Carbon Matrix: An Ultrafast Na-Storage Cathode with the Potential of Outperforming Li Cathodes. *Nano Letters* **14**, 2175-2180 (2014).

29. Fang, Y.J., Xiao, L.F., Ai, X.P., Cao, Y.L. & Yang, H.X. Hierarchical Carbon Framework Wrapped Na3V2(PO4)(3) as a Superior High-Rate and Extended Lifespan Cathode for Sodium-Ion Batteries. *Advanced Materials* **27**, 5895-5900 (2015).

30. Xu, Y.A. *et al.* Layer-by-Layer Na3V2(PO4)(3) Embedded in Reduced Graphene Oxide as Superior Rate and Ultralong-Life Sodium-Ion Battery Cathode. *Advanced Energy Materials* **6,** 1600389 (2016).

31. Yu, C.Y. *et al.* NaCrO2 cathode for high-rate sodium-ion batteries. *Energ Environ Sci* **8**, 2019-2026 (2015).

32. Fang, Y.J. *et al.* Mesoporous Amorphous FePO4 Nanospheres as High-Performance Cathode Material for Sodium-Ion Batteries. *Nano Letters* **14**, 3539-3543 (2014).

33. Tahir, M.N. *et al.* Extraordinary Performance of Carbon-Coated Anatase TiO2 as Sodium-Ion Anode. *Advanced Energy Materials* **6,** 1501489 (2016).

34. Luo, W. *et al.* Low-Surface-Area Hard Carbon Anode for Na-Ion Batteries via Graphene Oxide as a Dehydration Agent. *Acs Applied Materials & Interfaces* **7**, 2626-2631 (2015).

35. Wen, Y. *et al.* Expanded graphite as superior anode for sodium-ion batteries. *Nature Communications* **5,** 4033(2014).

36. Kim, H. *et al.* Sodium Storage Behavior in Natural Graphite using Ether-based Electrolyte Systems. *Advanced Functional Materials* **25**, 534-541 (2015).

37. He, M., Kraychyk, K., Walter, M. & Kovalenko, M.V. Monodisperse Antimony Nanocrystals for High-Rate Li-ion and Na-ion Battery Anodes: Nano versus Bulk. *Nano Letters* **14**, 1255-1262 (2014).

38. Fan, L. *et al.* Electrochemical performance of rod-like Sb-C composite as anodes for Li-ion and Na-ion batteries. *Journal of Materials Chemistry A* **3**, 3276-3280 (2015).

39. Coelho, J. *et al.* Lithium Titanate/Carbon Nanotubes Composites Processed by Ultrasound Irradiation as Anodes for Lithium Ion Batteries. *Sci Rep* **7**, 7614 (2017).

40. Zheng, M. *et al.* MoS2 intercalated p-Ti3C2 anode materials with sandwich-like three dimensional conductive networks for lithium-ion batteries. *J Alloy Compd* **735**, 1262-1270 (2018).

41. Yin, Z. *et al.* Copper nanowire/multi-walled carbon nanotube composites as all-nanowire flexible electrode for fast-charging/discharging lithium-ion battery. *Nano Res* **11**, 769-779 (2018).

42. Wang, L. *et al.* Carbon nanotube decorated NaTi2(PO4)(3)/C nanocomposite for a high-rate and low-temperature sodium-ion battery anode. *Rsc Adv* **6**, 70277-70283 (2016).

43. Wu, Y.T., Nie, P., Wu, L.Y., Dou, H. & Zhang, X.G. 2D MXene/SnS2 composites as high-performance anodes for sodium ion batteries. *Chem Eng J* **334**, 932-938 (2018).

44. Zhang, B. *et al.* Percolation threshold of graphene nanosheets as conductive additives in Li4Ti5O12 anodes of Li-ion batteries. *Nanoscale* **5**, 2100-2106 (2013).

45. Tang, R. *et al.* How a very trace amount of graphene additive works for constructing an efficient conductive network in LiCoO2-based lithium-ion batteries. *Carbon* **103**, 356-362 (2016).

46. Liu, T. *et al.* Effects of graphene with different sizes as conductive additives on the electrochemical performance of a LiFePO4 cathode. *Rsc Adv* **7**, 20882-20887 (2017).

47. Bauer, W., Notzel, D., Wenzel, V. & Nirschl, H. Influence of dry mixing and distribution of conductive additives in cathodes for lithium ion batteries. *J Power Sources* **288**, 359-367 (2015).

48. Logan, E.R. *et al.* A Study of the Physical Properties of Li-Ion Battery Electrolytes Containing Esters. *Journal of The Electrochemical Society* **165**, A21-A30 (2018).

49. Arora, P. & Zhang, Z.M. Battery separators. *Chem Rev* **104**, 4419-4462 (2004).

50. Jiang, F.M. & Peng, P. Elucidating the Performance Limitations of Lithium-ion Batteries due to Species and Charge Transport through Five Characteristic Parameters. *Scientific Reports* **6** (2016).

51. Ehrl, A., Landesfeind, J., Wall, W.A. & Gasteiger, H.A. Determination of Transport Parameters in Liquid Binary Lithium Ion Battery Electrolytes I. Diffusion Coefficient. *Journal of the Electrochemical Society* **164**, A826-A836 (2017).

52. Ong, M.T. *et al.* Lithium Ion Solvation and Diffusion in Bulk Organic Electrolytes from First-Principles and Classical Reactive Molecular Dynamics. *Journal of Physical Chemistry B* **119**, 1535-1545 (2015).

53. Park, M., Zhang, X.C., Chung, M.D., Less, G.B. & Sastry, A.M. A review of conduction phenomena in Li-ion batteries. *J Power Sources* **195**, 7904-7929 (2010).

54. Ding, N. *et al.* Determination of the diffusion coefficient of lithium ions in nano-Si. *Solid State Ion.* **180**, 222-225 (2009).

55. He, H.W., Xiong, R. & Fan, J.X. Evaluation of Lithium-Ion Battery Equivalent Circuit Models for State of Charge Estimation by an Experimental Approach. *Energies* **4**, 582-598 (2011).

56. Bauhofer, W. & Kovacs, J.Z. A review and analysis of electrical percolation in carbon nanotube polymer composites. *Composites Science and Technology* **69**, 1486-1498 (2009).

57. Celzard, A., Mareche, J.F., Furdin, G. & Puricelli, S. Electrical conductivity of anisotropic expanded graphite-based monoliths. *Journal of Physics D-Applied Physics* **33**, 3094-3101 (2000).

58. Ling, Z. *et al.* Quantifying the Role of Nanotubes in Nano:Nano Composite Supercapacitor Electrodes. *Advanced Energy Materials* **8**, 1702364 (2018).
